# Supplementary material for: HDAC3 is a molecular brake of the metabolic switch supporting white adipose tissue browning
Source: Nat Commun. 2017 Jul 21;8:93. doi: 10.1038/s41467-017-00182-7 (PMC5522415; doi:10.1038/s41467-017-00182-7)
Supplement: Supplementary file 1 — Supplementary Information [file 41467_2017_182_MOESM1_ESM.pdf]

File Name: Supplementary Information
Description: Supplementary Figures and Supplementary Tables

File Name: Peer Review File

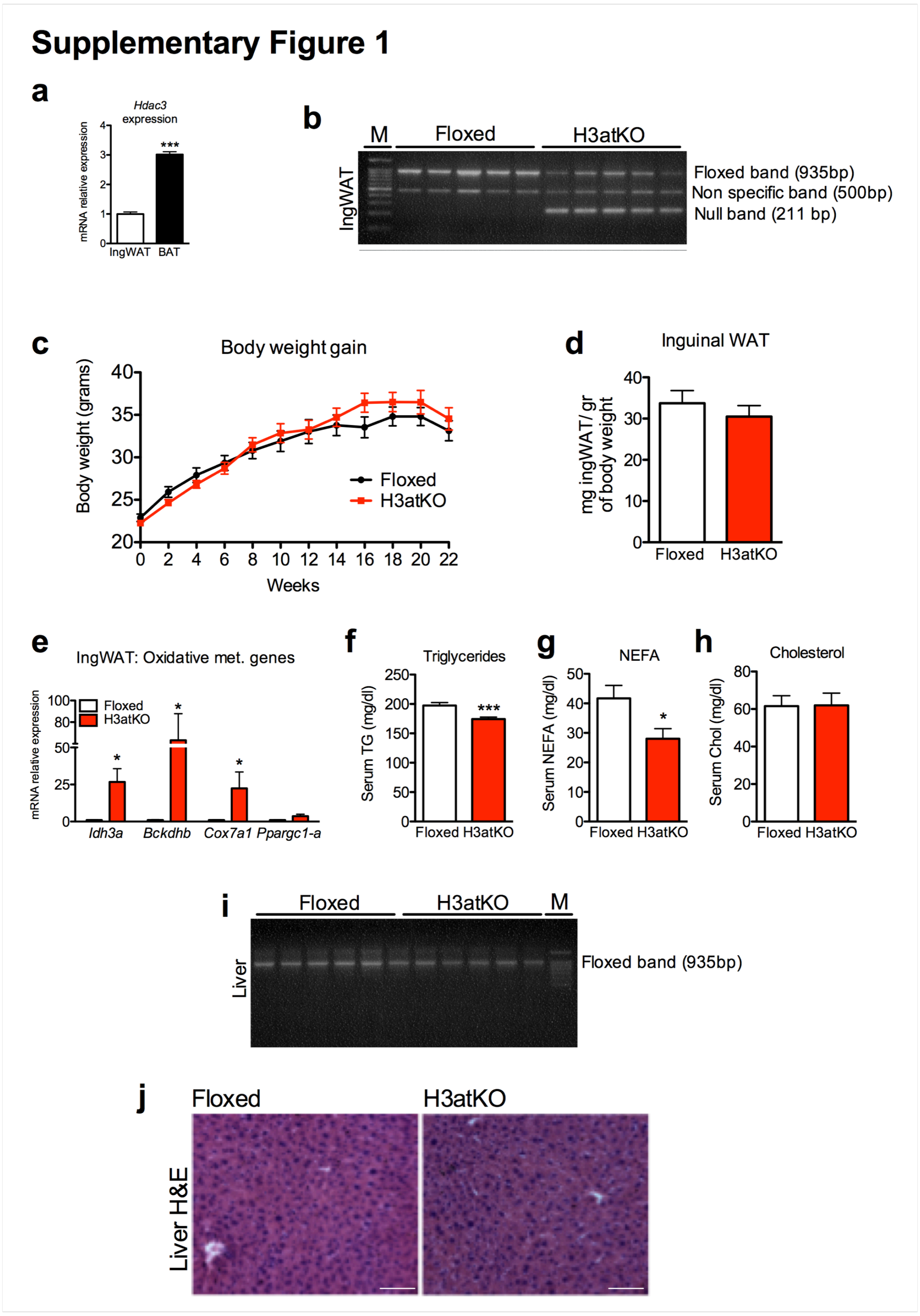

Supplementary Figure 1. Impact of *Hdac3* KO on inguinal WAT, serum lipids and liver

a) Hdac3 expression in Inguinal WAT and BAT; **b**) Deletion of *Hdac3* in IngWAT detected by PCR; **c**) Body weight gain of floxed and H3atKO mice (n=11 per group); **d**) Inguinal fat weight of floxed and H3atKO mice (n=11 per group); **e**) Gene expression analysis of oxidative metabolism genes in inguinal WAT of floxed and H3atKO mice (n=9-11 per group); **f, g, h**) Fasting levels of trigylcerides, cholesterol and non-esterified fatty acids in serum from floxed and H3atKO mice (n=11 per group); **i**) *Hdac3* in liver (negative control for deletion) of floxed and H3atKO mice detected by PCR; **j**) Hematoxylin and eosin staining of livers from Hdac3 floxed and knock out mice fed LFD, scale bar is 100μm.

Data are presented as mean ± SEM. Statistical analysis: Student's t test, $*p<0.05$, $***p<0.001$.

Supplementary Figure 2

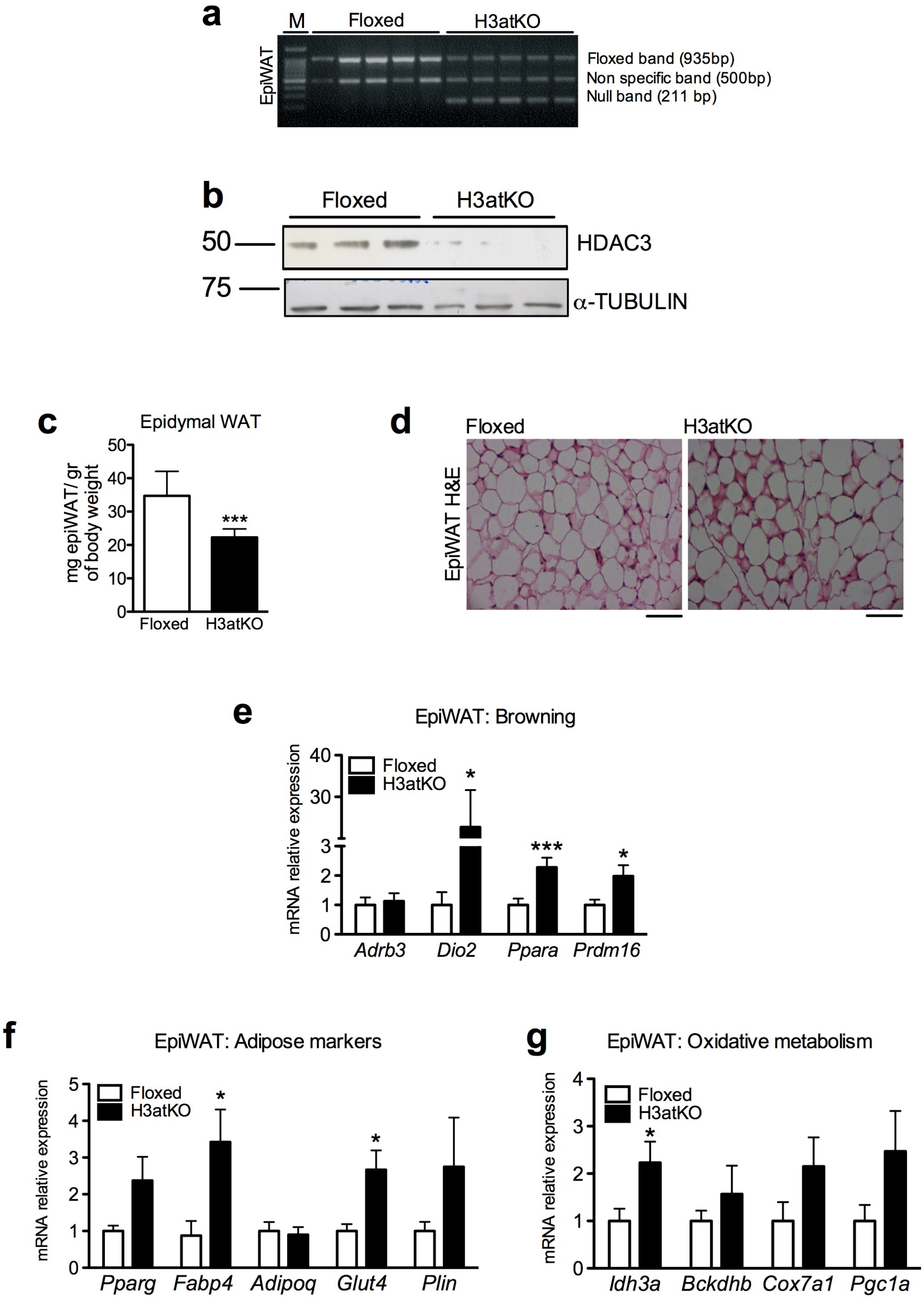

Supplementary Figure 2. Impact of *Hdac3* KO on epididymal WAT

a) Deletion of *Hdac3* in EpiWAT detected by PCR; **b**) Western blot analysis of HDAC3, showing the deletion of the protein in epididymal WAT of knock out mice versus floxed mice; **c**) Epididymal fat weight of floxed and H3atKO mice (n=11 per group); **d**) Hematoxylin-eosin staining of EpiWAT from floxed and H3atKO mice, scale bar is 100μm; **e**,**f**,**g**) Gene expression analysis of adipose markers, oxidative metabolism genes and browning genes in EpiWAT of floxed and H3atKO mice (n=9-11 per group).

Data are presented as mean ± SEM. Statistical analysis: Student's t test, *p<0.05, ***p<0.001.

Supplementary Figure 3

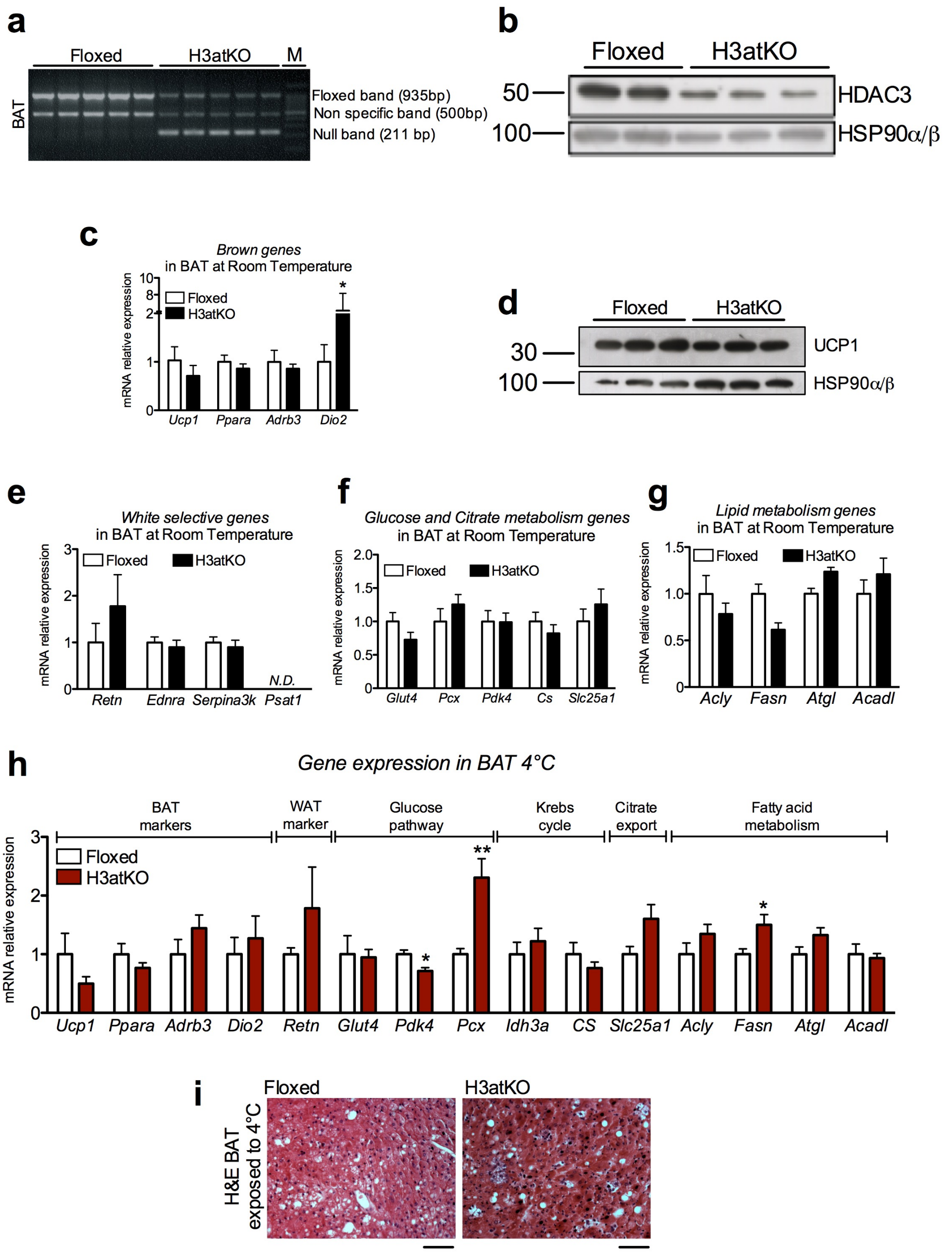

Supplementary Figure 3. Impact of *Hdac3* KO on BAT

a) Deletion of *Hdac3* in BAT detected by PCR; **b**) Western blot analysis of HDAC3, showing the deletion of the protein in BAT of knock out mice versus floxed mice; **c**) Gene expression analysis of brown genes in BAT of floxed and H3atKO mice (n=9-11 per group) housed at room temperature; **d**) Western blot showing UCP1 expression in BAT of floxed and H3atKO mice housed at room temperature; **e**, **f**, **g**) Gene expression analysis of white selective genes, glucose and citrate metabolism and lipid metabolism markers in BAT of floxed and H3atKO mice (n=9-11 per group) housed at room temperature; **h**) Gene expression analysis in BAT of floxed and H3atKO mice (n=6 per group) housed at 4°C for 24 hours; **i**) Hematoxylin-eosin staining of BAT from floxed and H3atKO mice housed at 4°C for 24 hours, scale bar is 100µm.

Data are presented as mean ± SEM. Statistical analysis: Student's t test, *p<0.05, **p<0.01.

Supplementary Figure 4

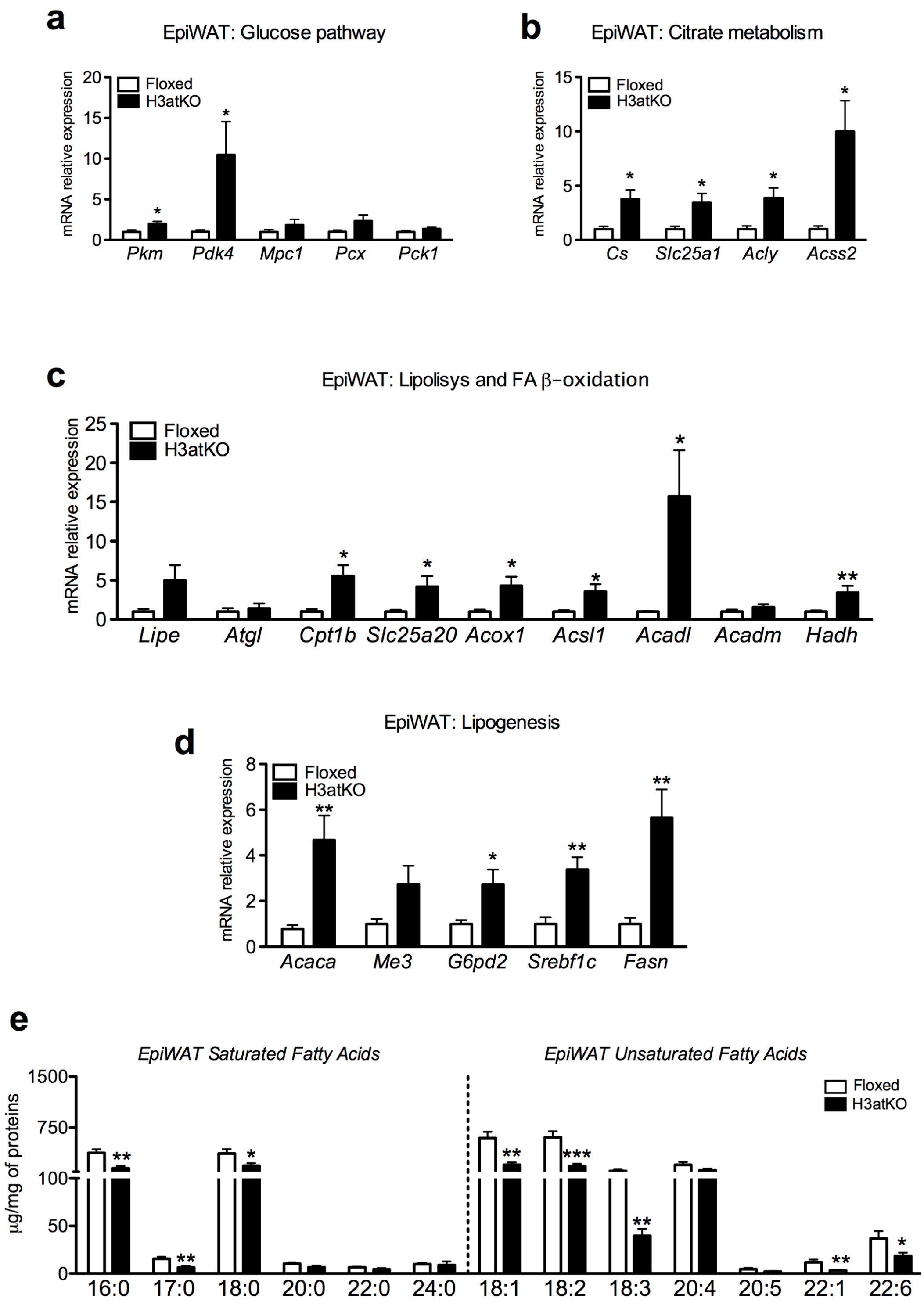

Supplementary Figure 4. Gene expression and fatty acid profile in epididymal WAT

a, **b**, **c**, **d**) Gene expression analysis of genes involved in glucose and citrate metabolism, lipolysis and FA β-oxidation, and lipogenesis in EpiWAT of floxed and H3atKO mice (n=9-11 per group); **e**) EpiWAT fatty acid quantification by mass spectrometry in floxed and H3atKO mice (n=8 per group).

Data are presented as mean ± SEM. Statistical analysis: Student's t test, *p<0.05, **p<0.01, ***p<0.001.

Supplementary Figure 5

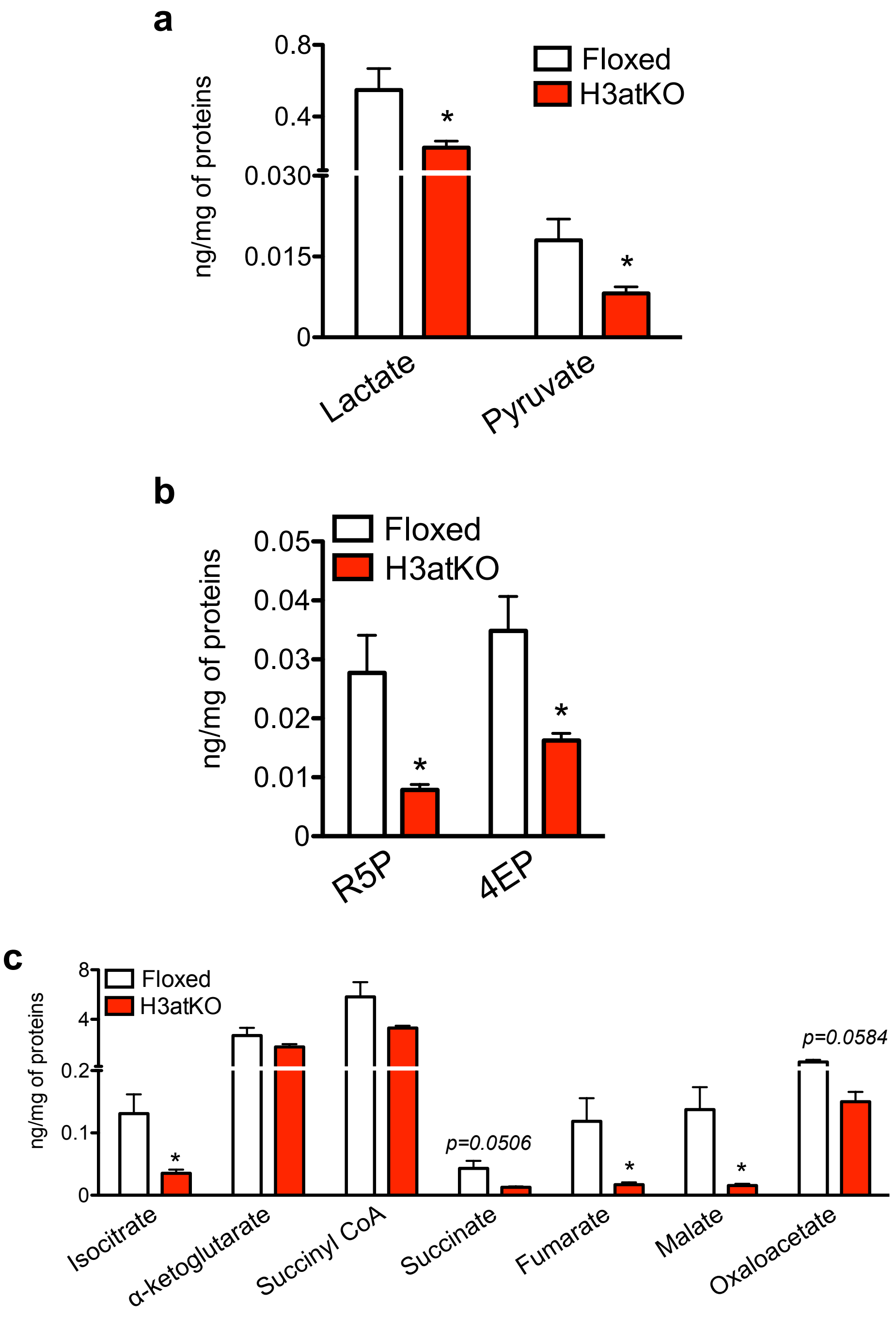

Supplementary Figure 5. Glucose and TCA cycle metabolites in inguinal WAT
a,**b**,**c**) Levels of lactate and pyruvate, pentose phosphate and TCA cycle intermediates in IngWAT of floxed and H3atKO mice (n=7-8 per group).
Data are presented as mean ± SEM. Statistical analysis: Student's t test, *$p<0.05$.

Supplementary Figure 6

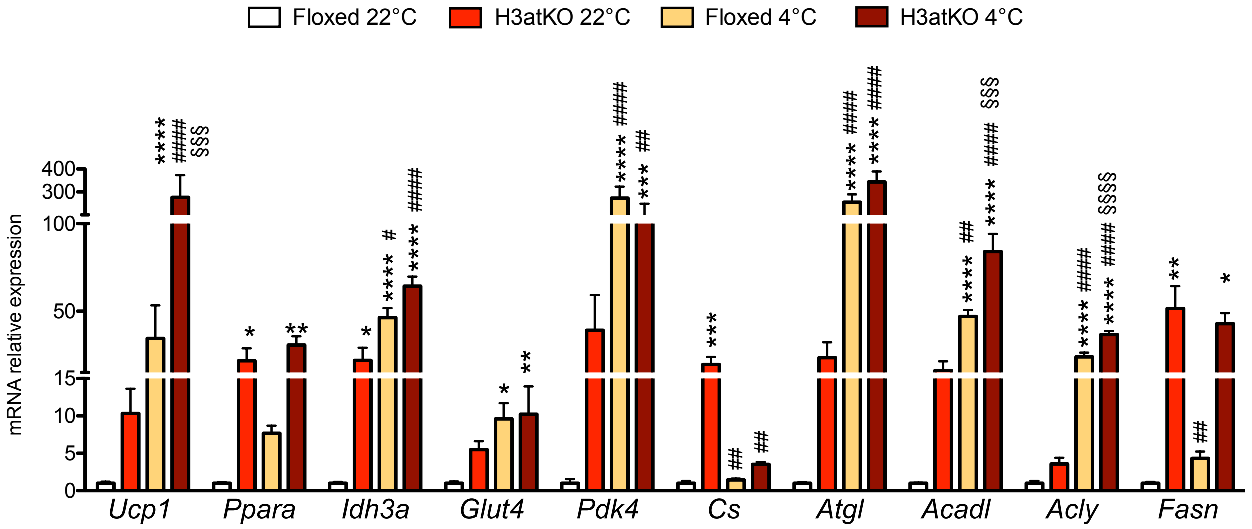

Supplementary Figure 6. Effect of temperature on gene expression in inguinal WAT
Gene expression analysis of IngWAT from floxed and H3atKO mice exposed to room temperature (22°C) or to 4°C fr 24 hours. Data are presented as mean ± SEM. Statistical analysis: Two way ANOVA, Tukey as post hoc test, *p<0.05, **p<0.01, ***p<0.001, ****p<0.0001 vs Floxed 22°C, #p<0.05, ##p<0.01, ###p<0.001, ####p<0.0001 vs H3atKO 22°C, §§§p<0.001, §§§§p<0.0001 vs Floxed 4°C.

Supplementary Figure 7

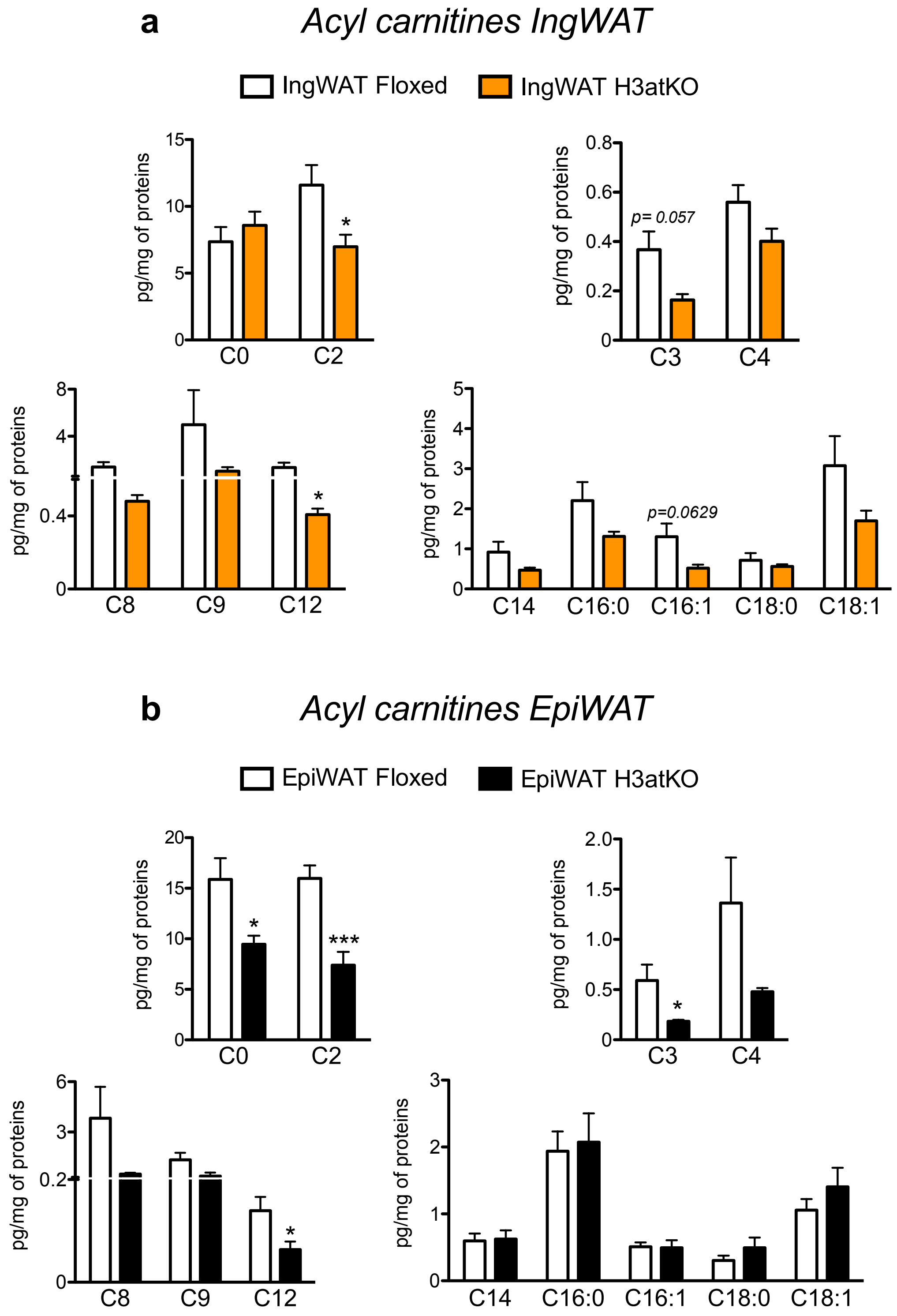

Supplementary Figure 7. Acyl carnitines in inguinal and epididymal WAT

a, **b**) Acylcarnitines levels in inguinal and epididymal WAT of *Hdac3* floxed and knock out mice (n=7-8 per group).

Data are presented as mean ± SEM. Statistical analysis: Student's t test, $*p<0.05$, $***p<0.001$.

Supplementary Figure 8

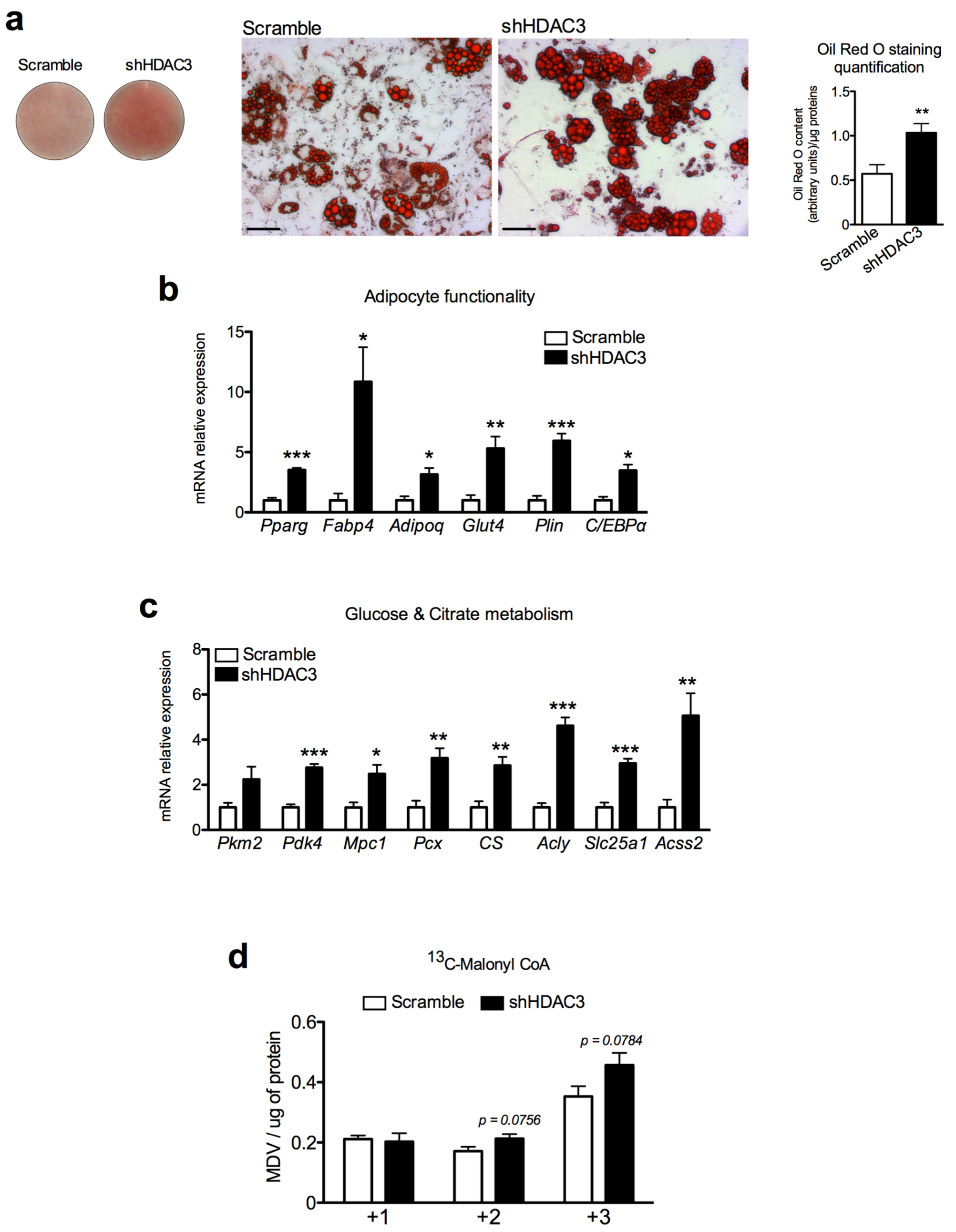

Supplementary Figure 8. HDAC3 silencing in cell culture recapitulates *in vivo Hdac3* KO

a) Oil Red O staining and quantification in C3H/10T1/2 cells infected with adenovirus expressing a shRNA targeted to *Hdac3* (shHDAC3) versus cell infected with a scrambled control shRNA (scramble) (n=10 per group), scale bar is 100µm; **b**, **c**) Gene expression analysis of genes involved in adipocyte functionality and in glucose and citrate metabolism in scramble and *Hdac3* knock down cells (n=4 biologicaln replicates per group); **d**) ^{13}C-labeled acetyl-CoA scramble and *Hdac3* knock down cells (n=6 per group).

Data are presented as mean ± SEM. Statistical analysis: Student's t test, *p<0.05, **p<0.01, ***p<0.001.

Supplementary Figure 9

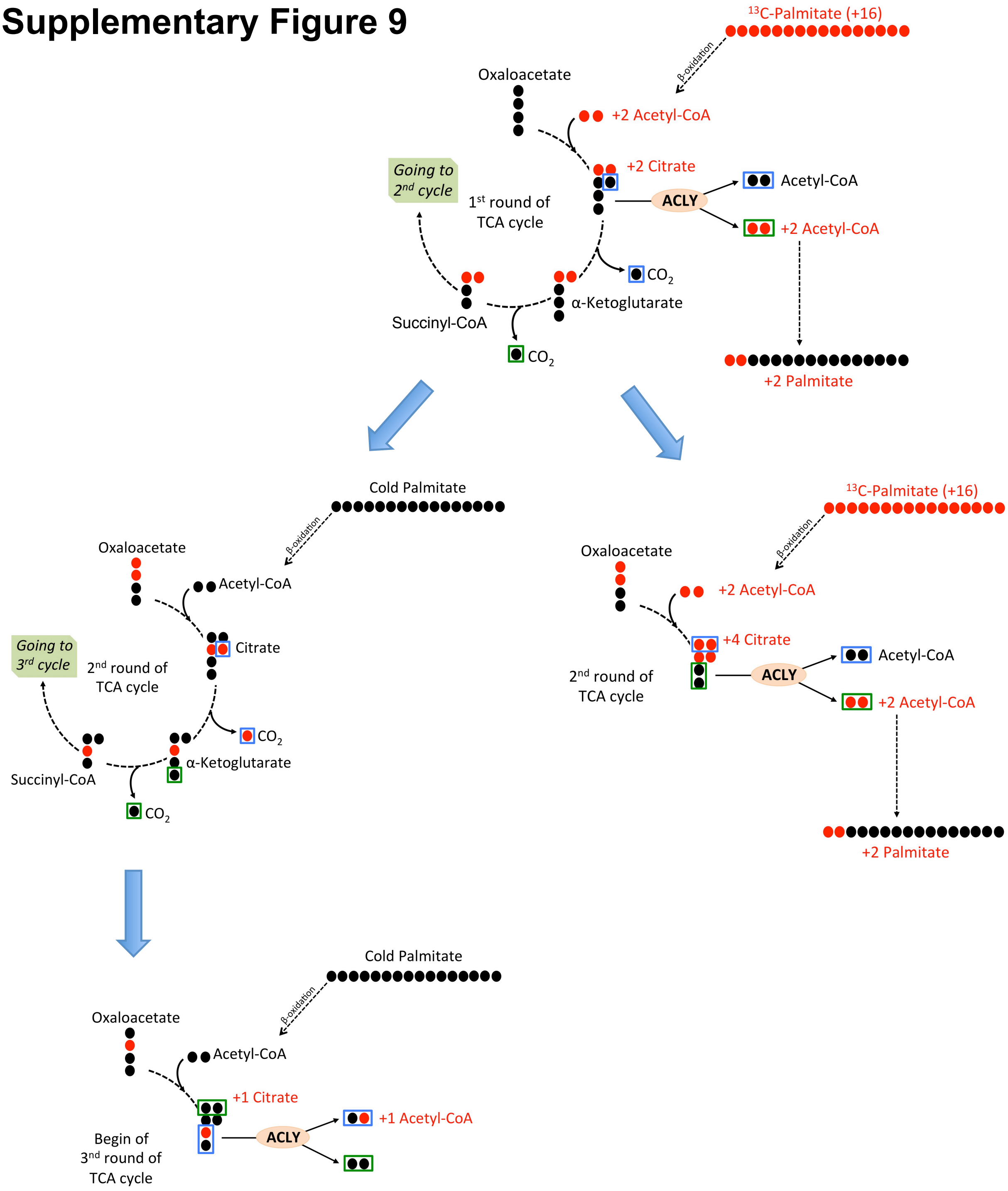

Supplementary Figure 9. Schematic representation of ^{13}C-palmitate incorporation into TCA cycle.
The first molecule of ^{13}C-palmitate undergoes β-oxidation and ^{13}C-labeled acetyl-CoA enters the cycle, and, condensing with oxaloacetate produces (+2) ^{13}C citrate. This citrate is in part used as substrate by ACLY enzyme, producing cold acetyl-CoA or (+2) ^{13}C Acetyl-CoA. (+2) ^{13}C citrate can also proceed into Krebs cycle, forming at the end of the first round (+2) ^{13}C oxaloacetate. If a molecule of cold acetyl-CoA (originating from endogenous cold palmitate) enters the cycle, at the end of the second round a (+1) ^{13}C oxaloacetate will be available. This, by condensate with another molecule of cold acetyl-CoA, will provide (+1) ^{13}C citrate, which serves as substrate for ACLY to produce (+1) ^{13}C acetyl-CoA. Alternatively, if after the first round of Krebs cycle, a molecule of (+2) ^{13}C acetyl-CoA condensate with (+2) ^{13}C oxaloacetate, (+4) ^{13}C citrate will be available, and ACLY enzyme will produce both cold acetyl-CoA and (+2) ^{13}C acetyl-CoA, which could be incorporated into newly synthetized fatty acids (e.g. (+2) ^{13}C palmitate).

Supplementary Figure 10

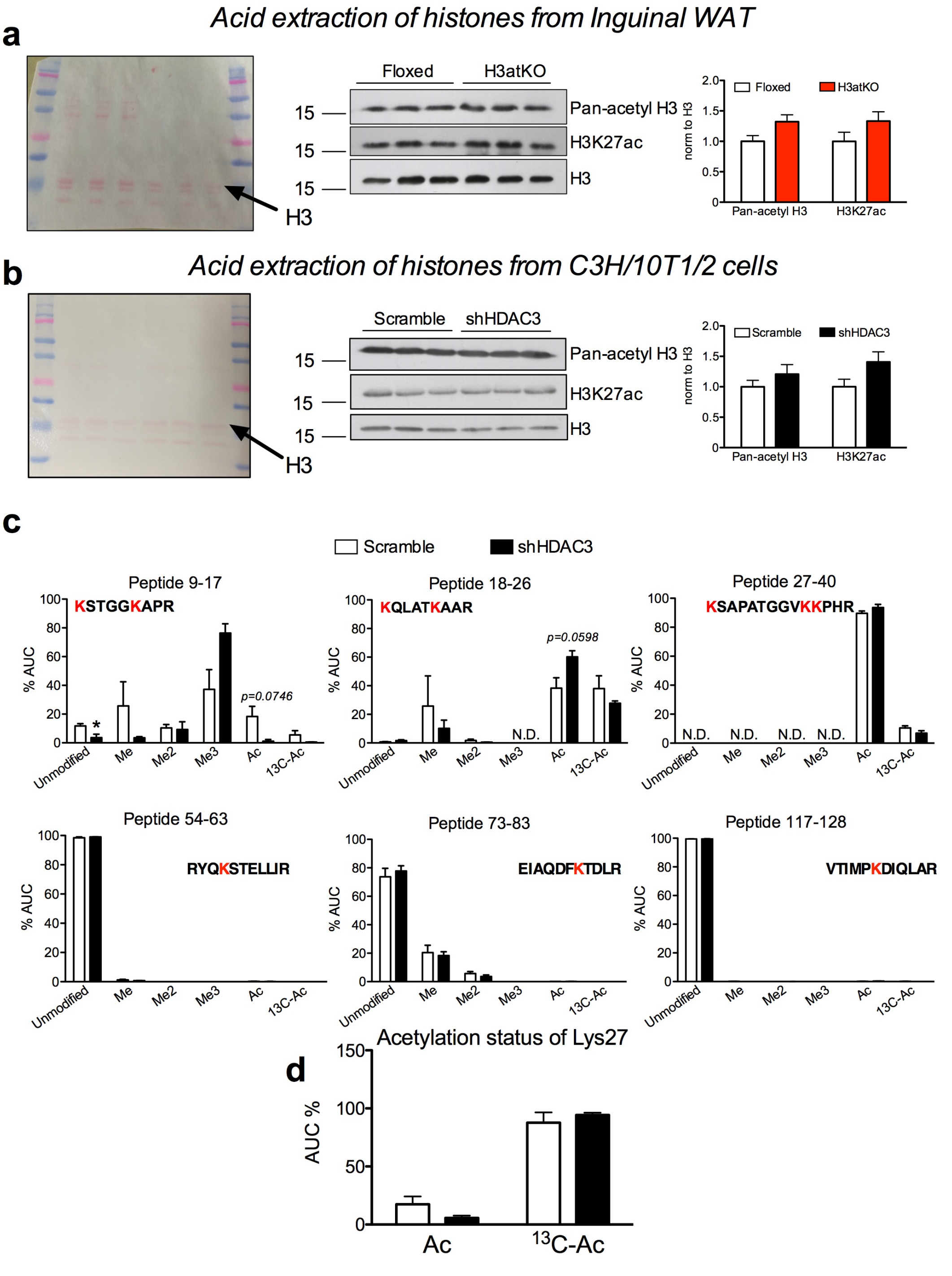

Supplementary Figure 10. Analysis of histone acetylation by western blot and mass spectrometry

a) Acid extraction of histones from Inguinal WAT: Ponceau S staining revealing the presence of histones, Western blot and quantification of and pan-acetyl H3 and H3K27ac in IngWAT from floxed and H3atKO mice; **b**) Acid extraction of histones from C3H10T1/2 cells: Ponceau S staining revealing the presence of histones, Western blot and quantification of and pan-acetyl H3 and H3K27ac in scramble and *Hdac3* knock down cells; **c**) Mass spectrometry analysis of histone H3 methylation/acetylation status in cells infected with scramble or HDAC3 shRNAs and incubated with [13C]-palmitate; **d**)Mass spectrometry analysis of histone H3(27-40) acetylation status of Lysine 27 in cells infected with scramble or HDAC3 shRNAs and incubated with [^{13}C]-palmitate.

Data are presented as mean ± SEM. Statistical analysis: Student's t test, *p<0.05.

Supplementary Figure 11

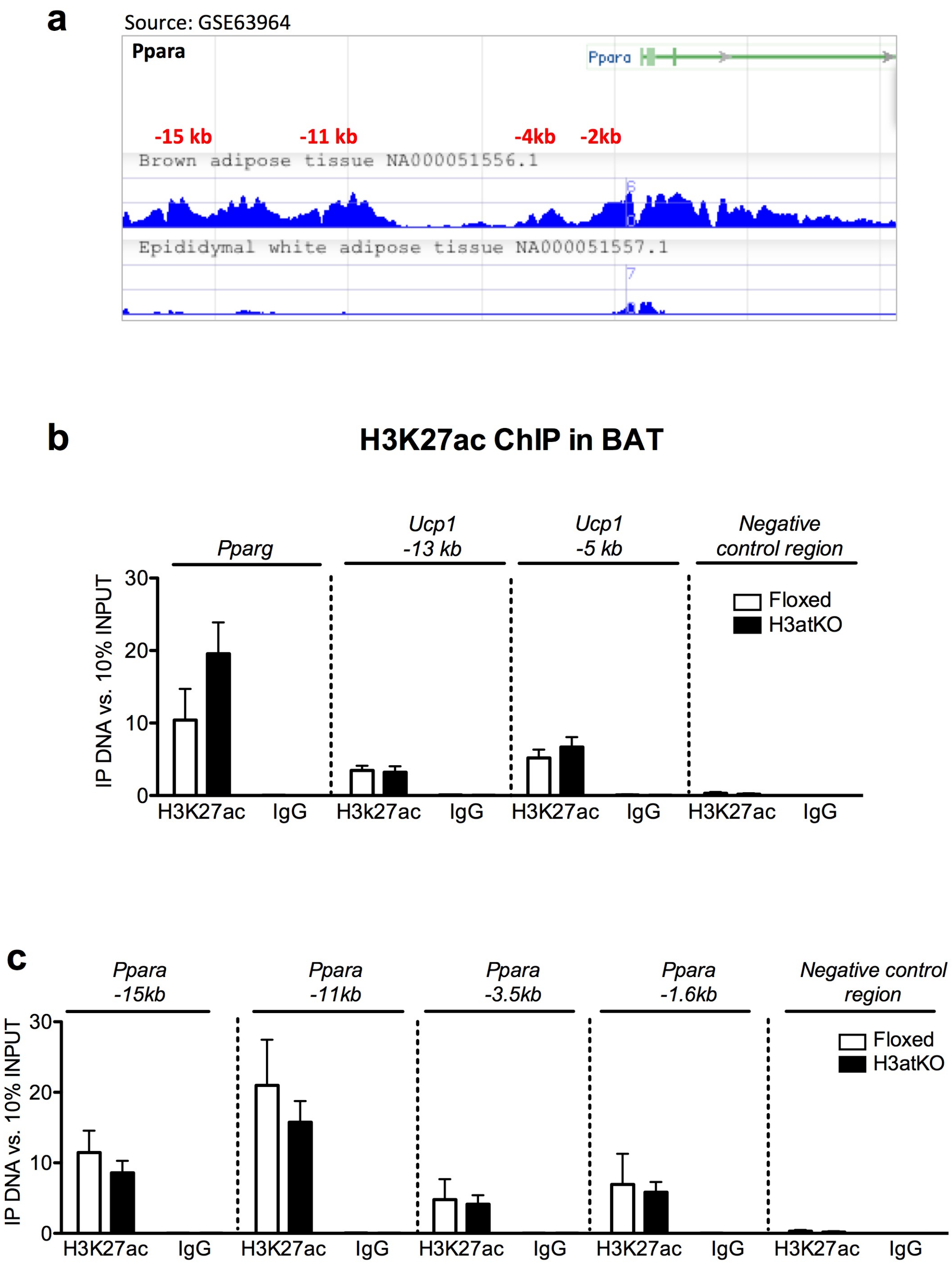

Supplementary Figure 11. Analysis of enhancer acetylation in BAT

a) H3K27ac ChIP-seq profile in BAT versus EpiWAT on upstream *Ppara* TSS (Source: GSE63964); **b**, **c**) H3K27ac ChIP analysis in BAT of floxed and H3atKO mice (n=3 per group, where each sample represent a pool from 2 individual mice).

Data are presented as mean ± SEM. Statistical analysis: Student's t test.

Supplementary Figure 12

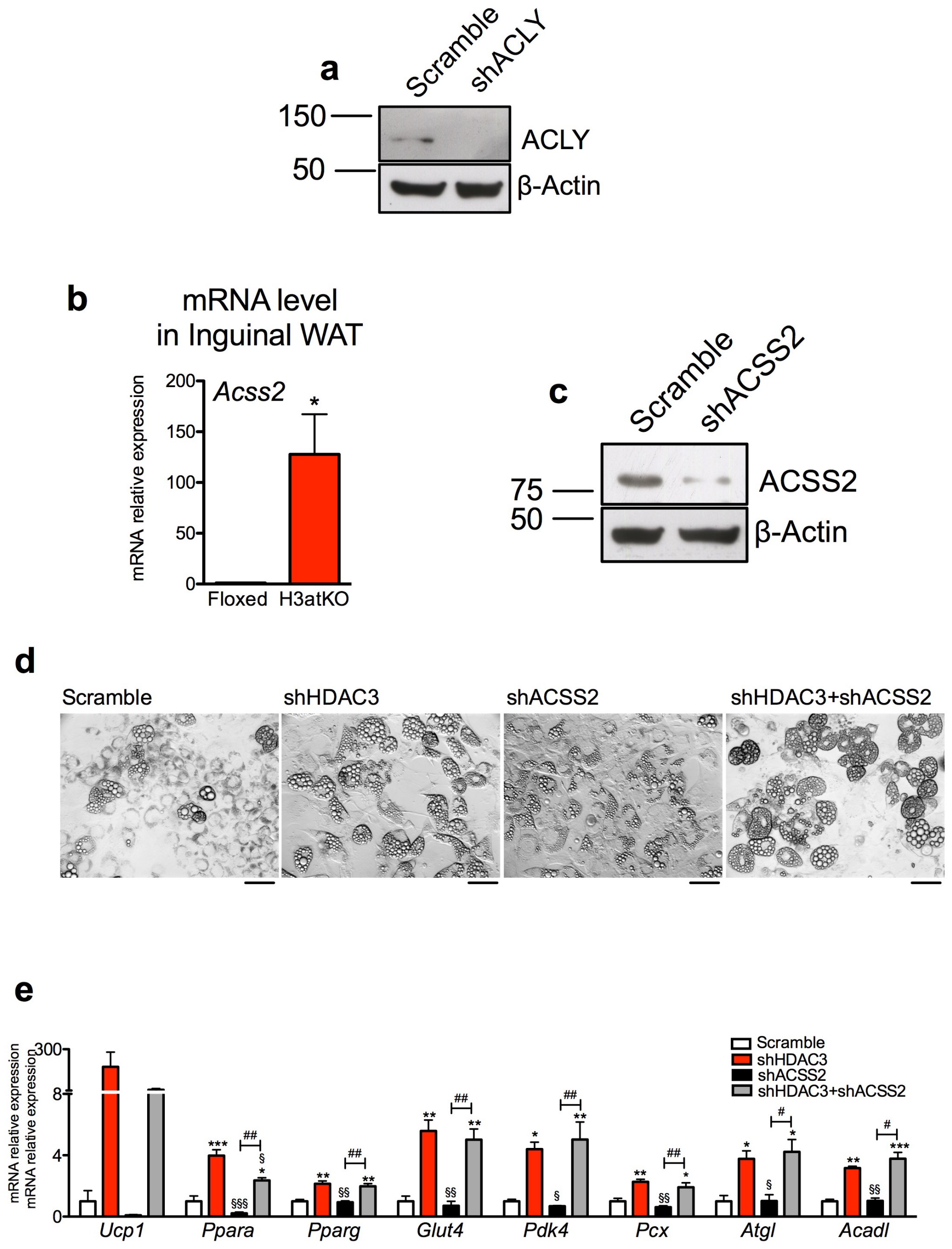

Supplementary Figure 12. Acss2 does not affect the phenotype of HDAC3 knocked down adipocytes
a) Efficiency of Acly silencing in cells infected with adenovirus expressing shRNA targeted to *Acly* (shACLY); **b**) Expression of *Acss2* gene in IngWAT of floxed and H3atKO mice (n=9-11 per group). Data are presented as mean ± SEM. Statistical analysis: Student's t test, *p<0.05, **p<0.01, ***p<0.001. **c**) Efficiency of Acss2 silencing in cells infected with adenovirus expressing shRNA targeted to *Acss2* (shACSS2); **d**) Morphology of cells infected with adenovirus expressing shRNA targeted to *Hdac3* (shHDAC3), to *Acss2* (shACSS2), or both (shHDAC3+shACSS2) or to a scrambled control shRNA (scramble), scale bar is 50μm; **e**) Gene expression analysis in knocked down cells (n=3 per group). Data are presented as mean ± SEM. Statistical analysis: 1 way ANOVA, Tukey as post hoc test, *p<0.05, **p<0.01, ***p<0.001 vs scramble, §p<0.05, §§p<0.01, §§§p<0.001 vs shHDAC3, $^{\#}$p<0.05, $^{\#\#}$p<0.01, $^{\#\#\#}$p<0.001 vs shACSS2.

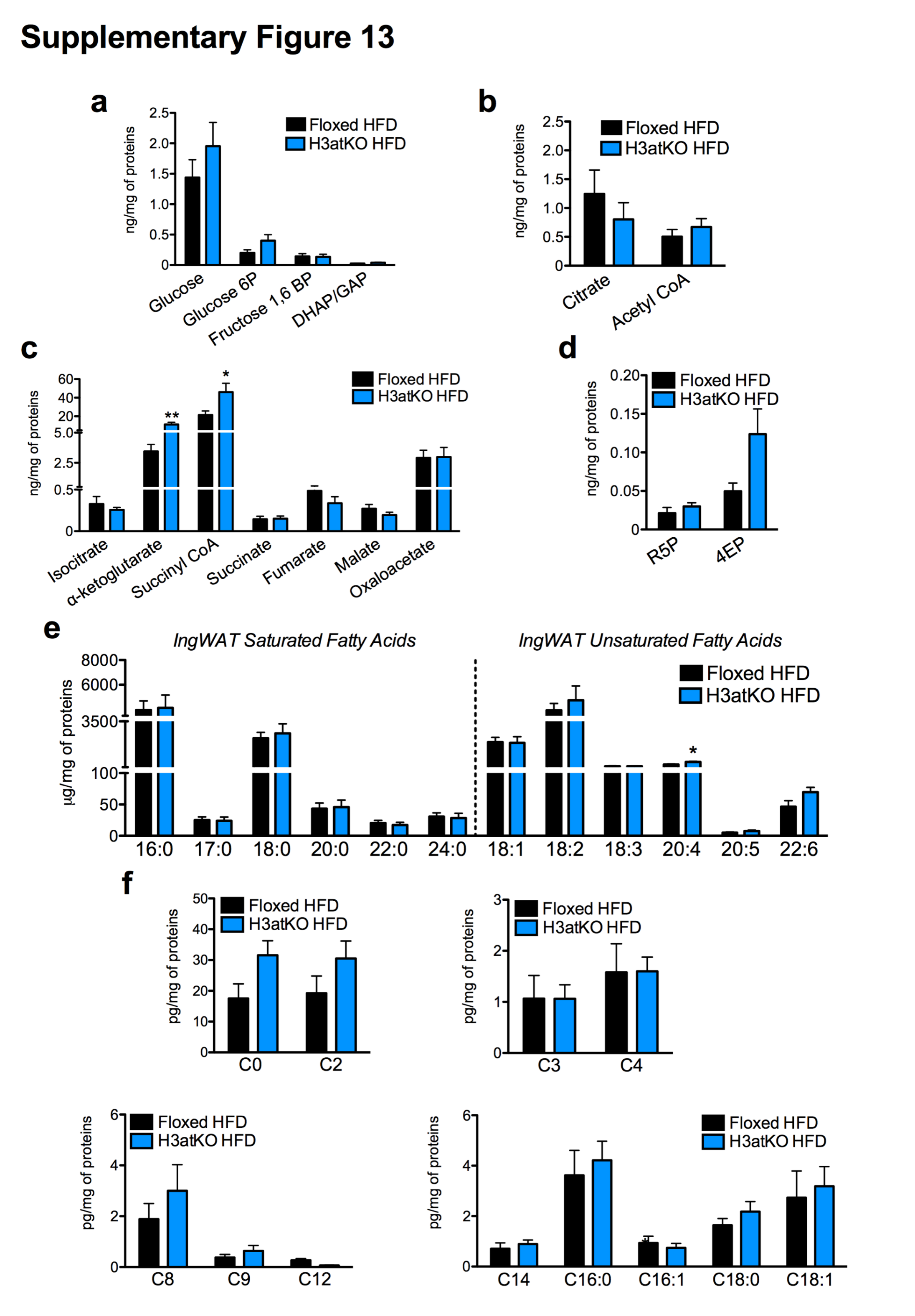

Supplementary Figure 13. High fat diet prevents the effects of *Hdac3* deletion

a, **b**, **c**, **d**) Levels of glycolytic intermediates, citrate, acetyl-CoA, TCA cycle and pentose phosphate intermediates in IngWAT from floxed and H3atKO mice fed HFD (n=6 per group); **e**) IngWAT fatty acid quantification by mass spectrometry in floxed and H3atKO mice (n=6 per group); **f**) Acylcarnitines levels in inguinal WAT of *Hdac3* floxed and knock out mice fed HFD (n=6 per group).

Data are presented as mean ± SEM. Statistical analysis: Student's t test, *p<0.05, **p<0.01.

Supplementary Figure 14

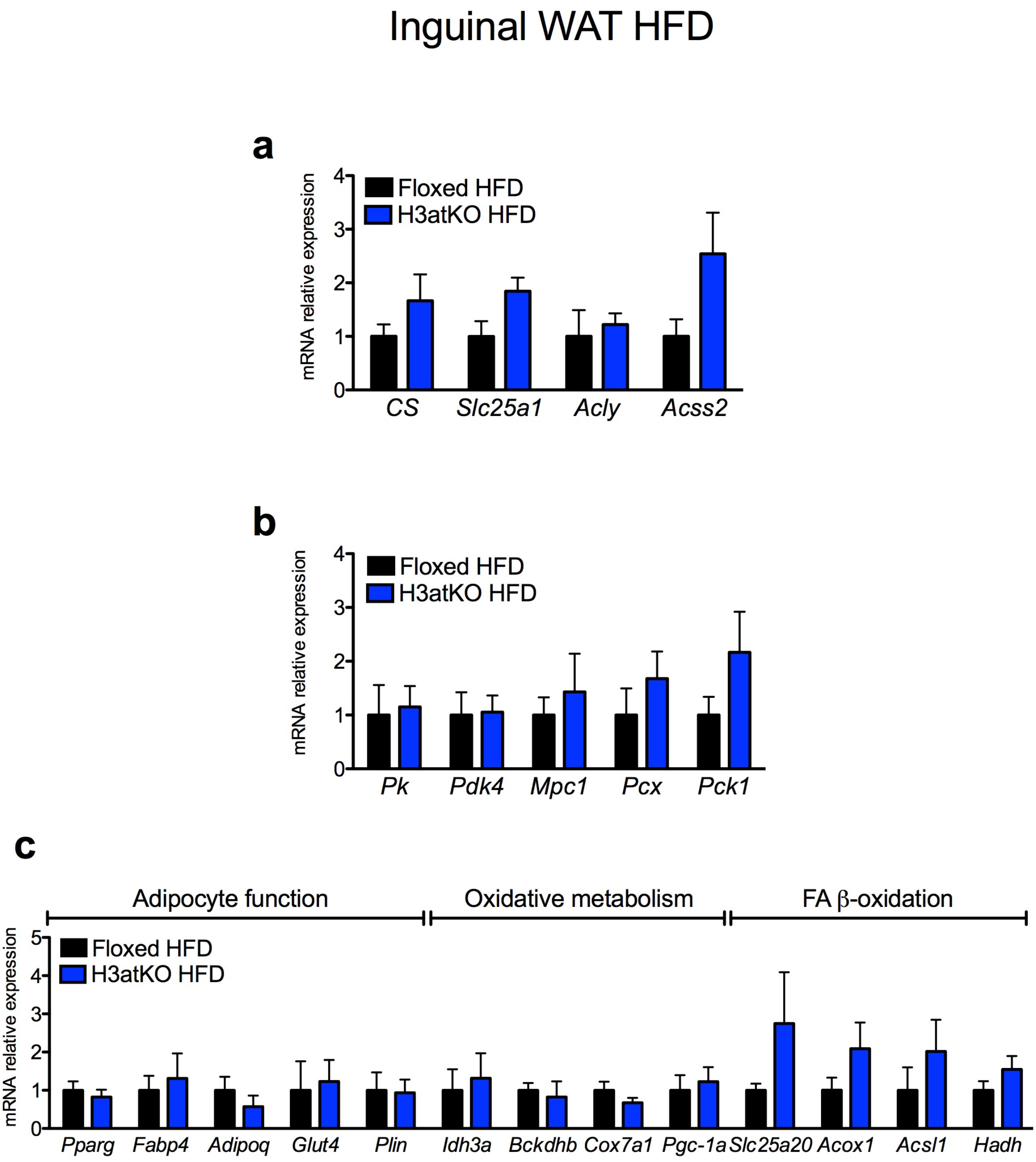

Supplementary Figure 14. Effect of *Hdac3* KO on gene expression in IngWAT of HFD fed mice.
a) Expression of genes related to citrate metabolism in IngWAT of floxed and H3atKO mice fed HFD (n=5 per group); **b**) Expression of genes related to glucose metabolism in IngWAT of floxed and H3atKO mice fed HFD (n=5 per group); **c**) Expression of genes involved in adipocyte functionality, oxidative metabolism and FA β-oxidation in IngWAT of floxed and H3atKO mice fed HFD (n=5 per group).
Data are presented as mean ± SEM. Statistical analysis: Student's t test.

Supplementary Figure 15

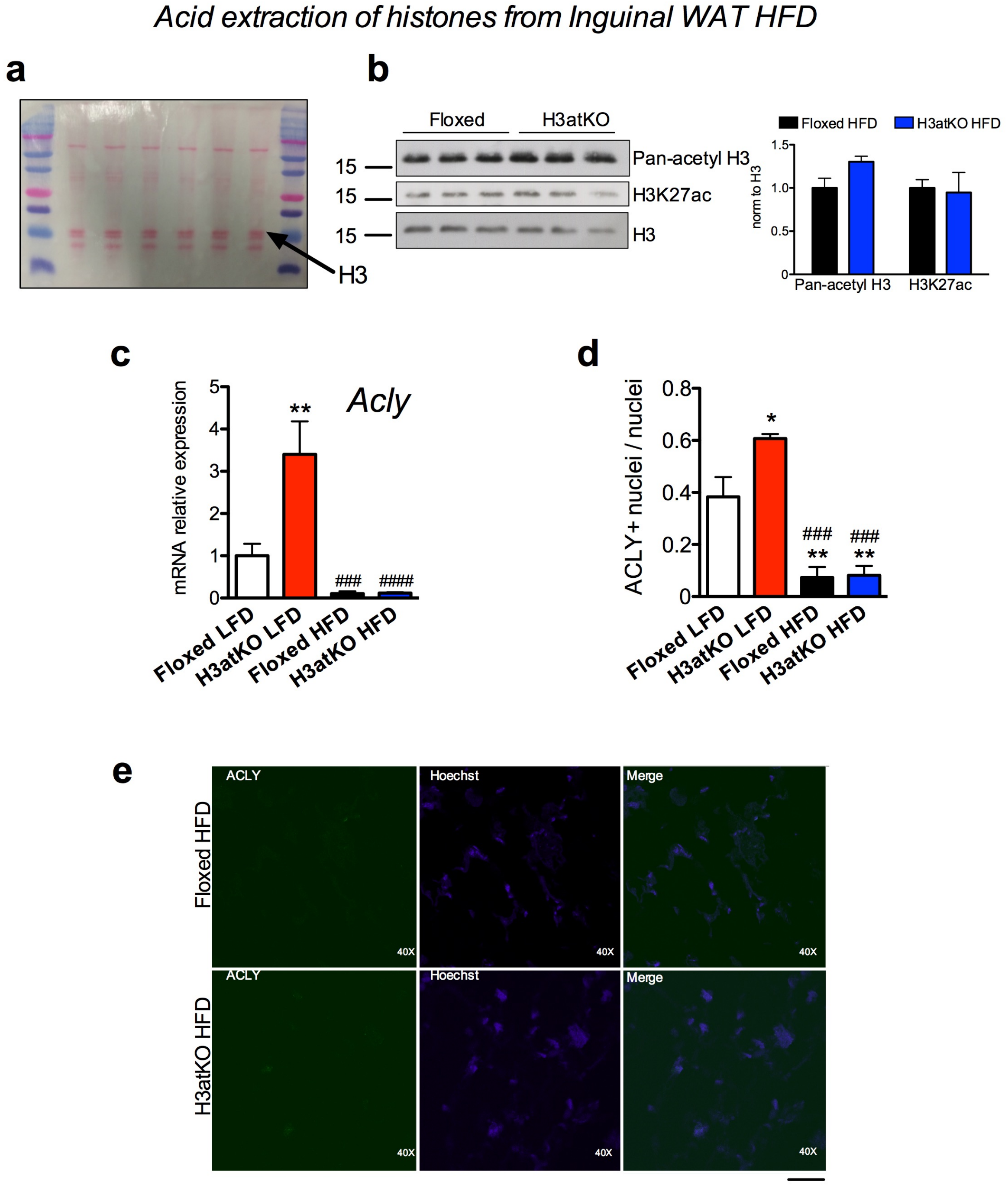

Supplementary Figure 15. Histone acetylation, *Acly* expression and localization in HFD mice

a, b) Acid extraction of histones from Inguinal WAT: Ponceau S staining revealing the presence of histones, Western blot and quantification of and pan-acetyl H3 and H3K27ac in IngWAT from floxed and H3atKO mice fed HFD. Data are presented as mean ± SEM. Statistical analysis: Student's t test, *p<0.05, **p<0.01, ***p<0.001; **c**) Expression of *Acly* gene in IngWAT of floxed and H3atKO mice fed LFD (n=9-11 per group) or fed HFD (n=12 per group); **d**, **e**) Immunofluorescence staining and quantification of ACLY, showing nuclear localization in *Hdac3* knock out mice fed LFD or HFD (n=3 per group), scale bar is 40 μm. Quantification of ACLY+ nuclei in samples from mice fed LFD was previously reported also in Fig 7b. Data are presented as mean ± SEM. Statistical analysis: Two way ANOVA, Tukey as post hoc test, *p<0.05, **p<0.01, vs Floxed LFD, $^{###}$p<0.001, $^{####}$p<0.0001 vs H3atKO LFD.

Supplementary Figure 16

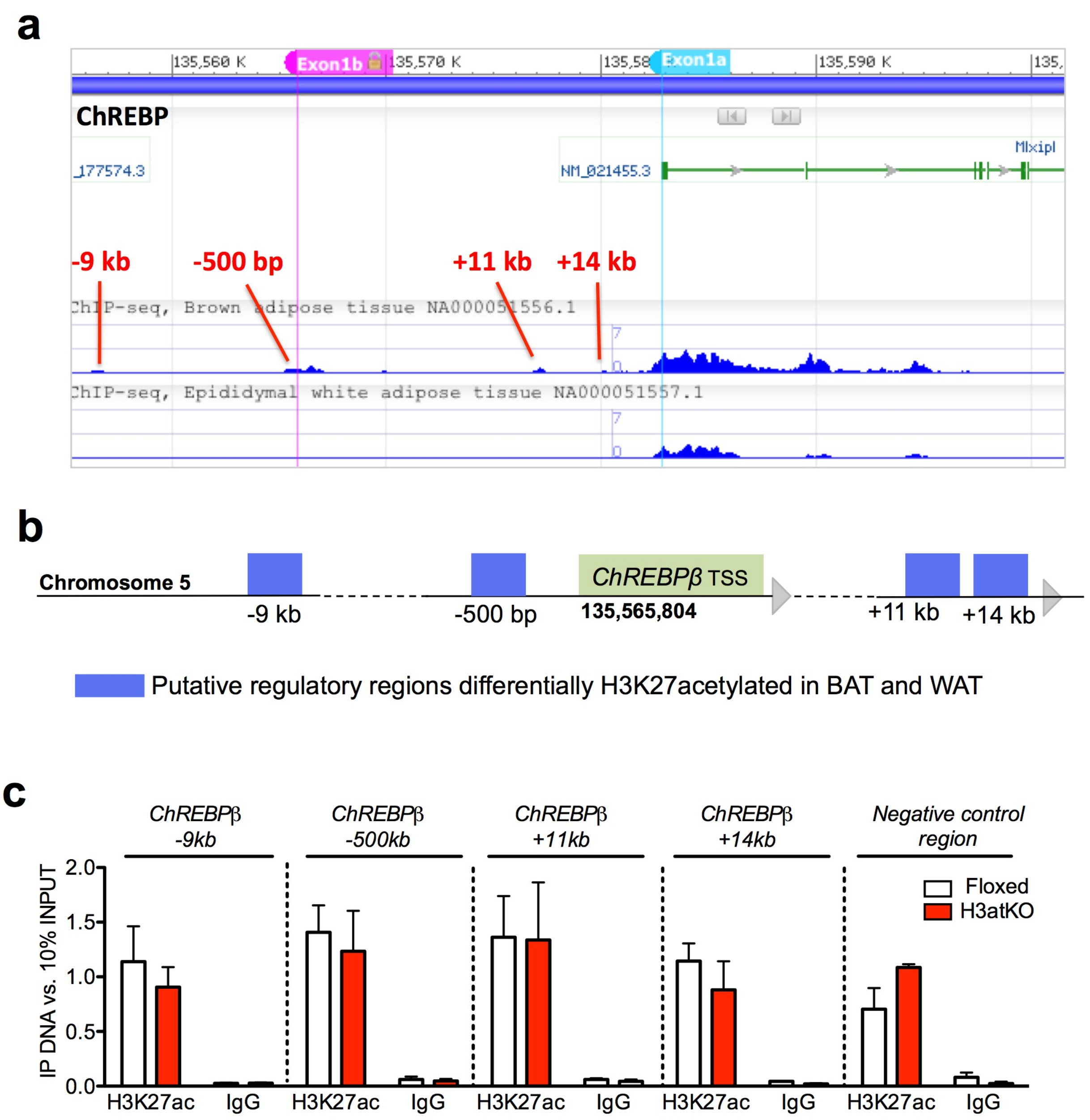

Supplementary Figure 16. Analysis of H3K27ac histone marks on *ChREBPβ* regulatory regions.

a, **b**) H3K27ac ChIP-seq profile in BAT versus EpiWAT on upstream *ChREBPβ* TSS (Source: GSE63964); **c**) H3K27ac ChIP analysis on regions upstream and downstream of *ChREBPβ* TSS in IngWAT from floxed and H3atKO mice fed LFD (n=3 per group, where each sample represent a pool from 2 individual mice).

Data are presented as mean ± SEM. Statistical analysis: Student's t test.

Supplementary Figure 17

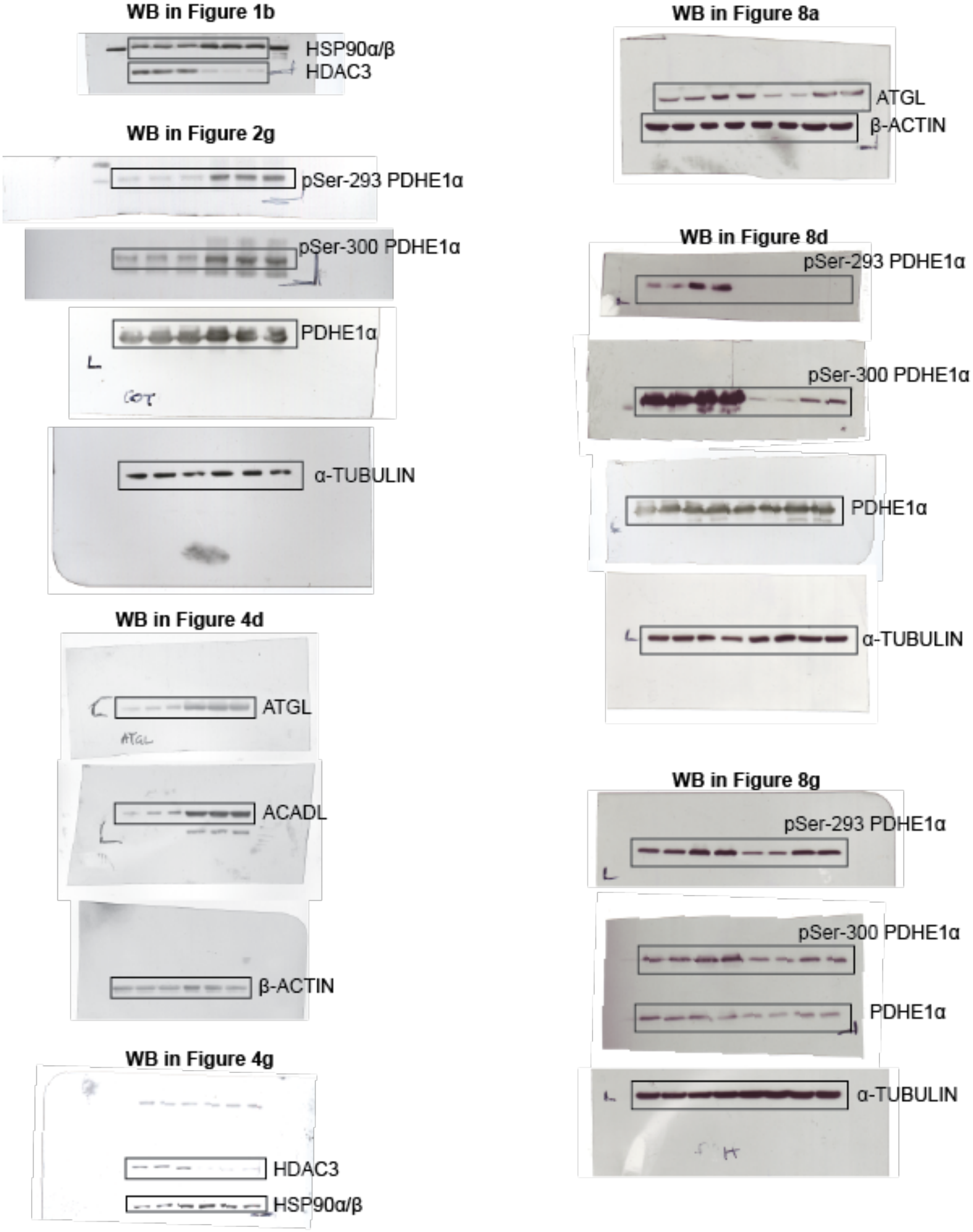

Supplementary Figure 17. Original films (uncropped scans) of western blot included in the main figures.

Supplementary Table 1

Volcano Plot of LFD mice

Volcano Plot LFD

	Log2 fold change	-Log10 p-value
Glucose	-1.563	1.415
G6P	-1.263	1.696
F1,6BP	-1.763	0.797
GAP	-1.886	1.627
Lactate	-1.278	1.433
Piruvate	-1.137	1.326
R5P	-1.815	1.685
E4P	-1.101	1.743
Acetyl-CoA	-1.146	1.424
Citrate	-3.356	3.358
Isocitrate	-1.899	1.686
2-Oxoglutarate	-0.6	0.675
Succinyl-CoA	-0.814	1.099
Succinate	-1.74	1.303
Fumarate	-2.815	1.475
Malate	-3.15	1.844
Oxoalacetate	-1.899	1.238
Malonyl-CoA	-1.312	1.584
NADH	-0.942	0.745
NADPH	-0.741	0.98
ATP	-0.093	0.092
ADP	-1.364	0.437
AMP	-1.154	0.513
Carnitine	0.223	0.365
Acetyl-L-carnitine	-0.732	1.616
Propionyl-L-carnitine	-0.878	0.939
Butyryl-L-carnitine	-0.483	1.075
Valeryl-L-carnitine	-0.358	0.547
Hexanoyl-L-carnitine	-1.991	0.898
Octanoyl-L-carnitine	-1.525	1.128
Nonayl-L-carnitine	-2.271	0.637
Decanoyl-L-carnitine	-1.723	1.182
Dodecanoyl-L-carnitine	-1.963	1.317
Tetradecanoyl-L-carnitine	-0.967	0.87
Palmitoyl-L-carnitine	-0.749	0.98
Hexadecenoyl-L-carnitine	-1.321	1.239
Stearoyl-L-carnitine	-0.346	0.349
Octadecenoyl-L-carnitine	-0.856	0.922
Palmitic acid	-1.16	1.678
Stearic acid	-1.153	1.774
Oleic acid	-1.446	1.835

Linoleic acid	-1.259	1.652
gamma-linolenic acid	-1.773	1.429
Arachidonic acid	-1.081	0.96
Docosahexaenoic acid	-1.421	1.246
Arachidic acid	-1.252	1.8
Behenic acid	-1.781	1.909
Erucic acid	-1.656	1.562
Lignoceric acid	-1.721	1.836
Margaric acid	-1.432	1.865

Supplementary Table 2

Volcano Plot of HFD mice

Volcano Plot HFD

	Log2 fold change	-Log10 p-value
Glucose	0.245	0.499
G6P	0.738	1.018
F1,6BP	-0.296	0.041
GAP	0.389	0.739
Lactate	0.209	0.392
Piruvate	0.019	0.086
R5P	0.229	0.46
E4P	1.054	1.124
Acetyl-CoA	0.241	0.389
Citrate	-0.603	0.396
Isocitrate	-0.349	0.317
2-Oxoglutarate	1.442	1.704
Succinyl-CoA	0.876	1.421
Succinate	-0.094	0.047
Fumarate	-0.55	0.557
Malate	-0.439	0.634
Oxoalacetate	-0.128	0.026
Malonyl-CoA	0.09	0.183
NADH	0.977	1.829
NADPH	0.455	0.72
ATP	0.321	0.442
ADP	0.912	1.136
AMP	0.614	0.93
Carnitine	0.59	1.205
Acetyl-L-carnitine	0.499	0.73
Propionyl-L-carnitine	-0.216	0.018
Butyryl-L-carnitine	-0.183	0.032
Octanoyl-L-carnitine	0.468	0.426
Nonayl-L-carnitine	0.506	0.524
Dodecanoyl-L-carnitine	-0.162	0.004

Tetradecanoyl-L-carnitine	0.296	0.356
Palmitoyl-L-carnitine	-0.18	0.18
Hexadecenoyl-L-carnitine	0.175	0.239
Stearoyl-L-carnitine	0.349	0.132
Octadecenoyl-L-carnitine	0.319	0.547
Palmitic acid	0.222	0.305
Stearic acid	0.054	0.175
Oleic acid	-0.164	0.029
Linoleic acid	0.115	0.267
gamma-linolenic acid	-0.179	0.043
Arachidonic acid	0.5	1.797
Docosahexaenoic acid	0.415	1.076
Arachidic acid	-0.053	0.062
Behenic acid	-0.316	0.216
Lignoceric acid	-0.244	0.081
Margaric acid	-0.191	0.056

Supplementary Table 3

PRIMERS for GENE EXPRESSION

Gene	Sequences
Pparg	ABI MIX Mm01184322_m1
Adrb3	ABI MIX Mm02601819_g1
Prdm16	ABI MIX Mm00712556_m1
36b4	Forward AGATGCAGCAGATCCGCAT<br>Reverse GTTCTTGCCCATCAGCACC<br>Probe CGCTCCGAGGGAAGGCCG
Ucp1	Forward GAGCTGGTAACATATGACCTC<br>Reverse GAGCTGACAGTAAATGGCA<br>Probe ACAAAATACTGGCAGATGACGTCCC
Dio2	Forward CTGTGTCTGGAACAGCTT<br>Reverse CACTGGAATTGGGAGCAT<br>Probe CTAGATGCCTACAAACAGGTTAAACTGGGT
Ppara	Forward ACGCATGTGAAGGCTGTAAG<br>Reverse CACTTGTGAAAACGGCAGTAC<br>Probe CGGCTGAAGCTGGTGTACGACAA
Glut4	Forward TGTCGCTGGTTTCTCCAACTG<br>Reverse CCATACGATCCGCAACATACTG<br>Probe ACCTGTAACTTCATTGTCGGCATGGGTTT
Pkm2	Forward CCATTCTCTACCGTCCTGTTG<br>Reverse TCCATGTAAGCGTTGTCCAG<br>Probe TCAGGTCCCTTTGTATCCAGAGCCA
Pdk4	Forward AGTGACTCAAAGACGGGAAAC<br>Reverse GTGTGAGGTTTAATTCTGGCG

	Probe ATCGACCCAAACTGTGATGTGGTAGC
Pcx	Forward GAGCTTATCCCGAACATCCC<br>Reverse TCCATACCATTCTCTTTGGCC<br>Probe CACGTTGTCAGGGTAGTTGGTGTAGC
Mpc1	Forward GACTTCCGGGACTATCTCATG<br>Reverse GTCAGAGAATAGCAACAGAGGG<br>Probe CAGAGATTATCAGTGGGCGGATGACTTT
CS	Forward GGGACTTGTGTATGAGACTTCG<br>Reverse AGCCAAAATAAGCCCTCAGG<br>Probe ACTGTAGCCTCGGAAACGGATGC
Slc25a1	Forward CAGAAGCAGTGGTAGTCGTG<br>Reverse TTCCCTTTAGCCCTTGTTCC<br>Probe ATGACCAGACTTCCTCCAACCCC
Acly	Forward CTGACCTTGCTGAACCCC<br>Reverse CCCGAGTATTCCCCGTAAT<br>Probe TCTTGGAGGTGTCAATGAACTGGCG
Srebf1c	Forward ATGGATTGCACATTTGAAGACATGCT<br>Reverse CCTGTGTCCCCTGTCTCAC<br>Probe CTTCCCGGGCCTGTTTGACGCCCCCTA
Fasn	Forward TCGTGATGAACGTGTACCGG<br>Reverse CGGGTGAGGACGTTTACAAAG<br>Probe TGCCTTCCGTCACTTCCAGTTAGAGCA
Acaca	Forward AAGGCTATGTGAAGGATGTGG<br>Reverse CTGTCTGAAGAGGTTAGGGAAG<br>Probe AGCTGAGGAAGTTGGCTATCCAGTG
Atgl	Forward TCGTGTTTCAGACGGAGA<br>Reverse CACATAGCGCACCCCT<br>Probe TGCAGACATTGGCCTGGATGAG
Lipe	Forward GCTCCCTTTCCCCGA<br>Reverse ATGCAGAGATTCCCACCT<br>Probe CACTGTGACCTGCTTGGTTCAACT
Acadl	Forward GAAACCAGGAACTACGTGAAG<br>Reverse GCTGTCCACAAAAGCTCT<br>Probe CACACATACAGACGGTGCAGCATA
Acadm	Forward ACCCAGATCCTAAAGTACCC<br>Reverse CGAAAGCAATTCCTCTGGTG<br>Probe TGGCCCATGTTTAGTTCCTTTTTTCCAA
Hadh	Forward TCTTGACTATGTTGGACTGGATAC<br>Reverse AAGGACTGGGCTGAAATAAGG<br>Probe CTTGGACGGGTGGCATGAAATGG
Cpt1b	Forward GATGCAGTTCCAGAGAATCC<br>Reverse CTTGTTCTTGCCAGAGCT<br>Probe TCTGCCCACTCTACCCTTCCTC
Pck1	Forward TTGAACTGACAGACTCGCCCT<br>Reverse TGCCCATCCGAGTCATGA

	Probe CCGCATGCTGGCCACCACA
Acsl1	Forward CTGTGGGATAAACTCATCTTCC<br>Reverse CCTTCATAGAACTGGCAGC<br>Probe CCACCCAGGCTCGACTGTATCTT
Acox1	Forward TCACGTTTACCCCGGC<br>Reverse CAAGTACGACACCATACCAC<br>Probe CATCAAGAACCTGGCCGTCTGC
Slc25a20	Forward TCTTTGGGTTTGGTCTGGG<br>Reverse ATTTGATCCGTTCTCCAGGG<br>Probe TCTCCAGAGGATGAACTTAGCTACCCAC
Me3	Forward CCTATCGTCTTTGCCCTGAG<br>Reverse AGTGAATGTCCTGCCATCTTC<br>Probe CCGAGGAATCTTTGCCAGTGGAAGT
Chrebpb	Forward CTGCAGATCGCGTGGAG<br>Reverse GCAACTTGAGGCCTTTGAAG<br>Probe CAAGCTGGTCTCTCCCAAGTGGAA
G6pd2	Forward AAGAATGAACGGTGGGATGG<br>Reverse GGAAGATGTCGCCTGGTATATC<br>Probe CGCAAAGCTGAAGTGAGACTGCAA
Cidea	Forward CACGCATTTCATGATCTTGG<br>Reverse CCTGTATAGGTCGAAGGTGA<br>Probe TTACTACCCGGTGTCCATTTCTGTCC
Elovl3	Forward TGCTTTGCCATCTACACG<br>Reverse CAGTGGACAAAGATGAGTGG<br>Probe TGAACTGGGAGACACGGCCTT
Cd36	Forward GCGACATGATTAATGGCACAG<br>Reverse GATCCGAACACAGCGTAGATAG<br>Probe CAACAAAAGGTGGAAAGGAGGCTGC
Lpl	Forward GCCATGACAAGTCTCTGAAG<br>Reverse CTTTCAAACACCCAAACAAGG<br>Probe AGTCTGGCTGACACTGGACAAACA
Idh3a	Forward ACGGAAGGAGAATACAGTGG<br>Reverse GTACTCGAAGGCAAACTCTG<br>Probe ACCCCATCAACGATCACATGCTCA
Bckdhb	Forward TGGGATGTGGATACAGTTTGC<br>Reverse TCGAGATATTGGAGCCTCTAGG<br>Probe AGTCGCCCGGTTTTGATCACAGA
Cox7a1	Forward TGTGGCAGAGAAGCAGAAG<br>Reverse AGCCCAAGCAGTATAAGCAG<br>Probe CGACAATGACCTCCCAGTACACTTGA
Ppargc1a	Forward CATTTGATGCACTGACAGATGGA<br>Reverse GTCAGGCATGGAGGAAGGAC<br>Probe CCGTGACCACTGACA ACGAGGCC
Adipoq	Forward AGGCATCCCAGGACATC<br>Reverse CCTGTCATTCCAACATCTCC

	Probe CCTTAGGACCAAGAAGACCTGCATCTC
Plin	Forward ACAGACACAGAGGGAGAGG<br>Reverse AGTGTTCTGCACGGTGTG<br>Probe AGGAGGAAGAAGAGTCCGAGGCT
Retn	Forward GGCTTAAATTGCTGGACAGTC<br>Reverse TCTATCCTTGCACACTGGC<br>Probe TGGCTCGTGGGACATTCGTGAAG
Edrna	Forward GTCTTGAACCTCTGTGCTCTC<br>Reverse GATCCCGATTCCTTGAACTCG<br>Probe CCACTGCTCTGTACCTGTCCACAC
Psat1	Forward TGGAACGGTGAACATTGTCC<br>Reverse GTCAGGTACGAAGTCAAACTCC<br>Probe AAGCACCTGGAACCTCAACCCG
Serpina3k	Forward CAGAAACCAAGAAACTGAGTGTG<br>Reverse GGAATGGCCTGTTGAAATGC<br>Probe ACGAATGCCACCAATAACCCCTGT
Acss2	Forward ACCAGTTAAGAGGCCATGTC<br>Reverse GTACAAGATGAAGAGTGGGTCC<br>Probe AGGCAGGAGACGAGTGTGAGC
Fabp4	Forward GGCGTGGAATTCGATGAA<br>Reverse GCTTGTCACCATCTCGTT<br>Probe TGATGCTCTTCACCTTCCTGTCGT
Cebpa	Forward AGAGCCGAGATAAAGCCAAAC<br>Reverse TCATTGTCACTGGTCAACTCC<br>Probe AGCACCTTCTGTTGCGTCTCCA
Vegfa2	Forward GGAATCCCAGAAACAACCCTAAT<br>Reverse GACTGGATTCGCCATTTTCTTATATC

Supplementary Table 4

PRIMERS for ChIP

Gene	Sequences
Pparg enhancer	Forward TGAGGCAGACAGGACTGAAAGTGG<br>Reverse TGGTGCCCATCTGGAAGGCTGC
Ucp1 -13kb	Forward GCAACCCTCTCCCATCAGTG<br>Reverse GCCTAACACCGTGCTTCTCA
Ucp1 -5kb	Forward TGCAACCCCTCACCTTTTAC<br>Reverse CTCCTTCCATCATCCCTTCA
Ppara -15kb	Forward AGTGAGCGCCATGATACCAC<br>Reverse ATGGCTGGGCCAGGATAATG
Ppara -11kb	Forward CAGGGTGGAGGGAAACTCTC<br>Reverse TGGCTTTACCTCCTTGGGAC
Ppara -3.5kb	Forward AAGCAGGGTGCTTTGACCTT

	Reverse TCTGCTAGGAAACTGCTGCC
Ppara -1.6kb	Forward GTAAGCAGCCTCCAGCCATT<br>Reverse CCTGAATGGCCCAGGGGATA
Negative control	Forward ACTGTGCCAAACTTTGAGGGAAGG<br>Reverse TAGGGTGGGAGATGAGTGAGTGAA

Supplementary Table 5

DAVID analysis

1354 annotated genes out of 1429				
**Annotation Cluster 1**	**Mitochondrion**	**Enrichment Score: 23.76055310378521**		
Category	Term	Count	%	PValue
UP_KEYWORDS	Mitochondrion	169	12.48153619	3.98E-34
GOTERM_CC_DIRECT	GO:0005739~mitochondrion	224	16.54357459	3.61E-27
UP_KEYWORDS	Transit peptide	89	6.573116691	1.66E-20
UP_SEQ_FEATURE	transit peptide:Mitochondrion	85	6.277695716	3.79E-16
\\				
**Annotation Cluster 2**	**Endoplasmic reticulum**	**Enrichment Score: 7.366942941152303**		
Category	Term	Count	%	PValue
UP_KEYWORDS	Metal binding	105	7.754800591	1.87E-09
GOTERM_CC_DIRECT	GO:0005783~endoplasmic reticulum	133	9.822747415	5.19E-08
GOTERM_CC_DIRECT	GO:0005789~endoplasmic reticulum membrane	79	5.834564254	8.17E-07
\\				
**Annotation Cluster 3**	**metal binding**	**Enrichment Score: 6.55765609288561**		
Category	Term	Count	%	PValue
UP_KEYWORDS	Metal-binding	282	20.82717873	4.83E-11
GOTERM_MF_DIRECT	GO:0046872~metal ion binding	287	21.19645495	5.33E-09
UP_KEYWORDS	Zinc-finger	146	10.78286558	8.23E-09
UP_KEYWORDS	Zinc	181	13.36779911	3.18E-08
GOTERM_MF_DIRECT	GO:0003676~nucleic acid binding	125	9.231905465	6.44E-08
SMART	SM00349:KRAB	48	3.545051699	5.78E-07
INTERPRO	IPR001909:Krueppel-associated box	48	3.545051699	1.58E-06
INTERPRO	IPR013087:Zinc finger C2H2-type/integrase DNA-binding domain	68	5.022156573	1.67E-05
SMART	SM00355:ZnF_C2H2	70	5.169867061	1.69E-05
INTERPRO	IPR007087:Zinc finger, C2H2	74	5.465288035	1.97E-05

INTERPRO	IPR015880:Zinc finger, C2H2-like	70	5.169867061	3.33E-05
\				
**Annotation Cluster 4**	**Protein transport**	**Enrichment Score: 5.223334786900093**		
Category	Term	Count	%	PValue
UP_KEYWORDS	Protein transport	64	4.726735598	8.44E-07
UP_KEYWORDS	Transport	159	11.74298375	1.62E-06
GOTERM_BP_DIRECT	GO:0015031~protein transport	66	4.874446086	7.46E-06
GOTERM_BP_DIRECT	GO:0006810~transport	153	11.29985229	1.25E-04
\				
**Annotation Cluster 5**	**Peroxisome**	**Enrichment Score: 4.832144318061019**		
Category	Term	Count	%	PValue
UP_KEYWORDS	Peroxisome	23	1.698670606	1.29E-07
GOTERM_CC_DIRECT	GO:0005777~peroxisome	27	1.994091581	1.44E-07
KEGG_PATHWAY	mmu04146:Peroxisome	19	1.403249631	2.86E-06
GOTERM_CC_DIRECT	GO:0005778~peroxisomal membrane	11	0.812407681	0.001656165
UP_SEQ_FEATURE	short sequence motif:Microbody targeting signal	9	0.664697194	0.007872572
\				
**Annotation Cluster 6**	**Fatty acid metabolism**	**Enrichment Score: 4.5588316711046275**		
Category	Term	Count	%	PValue
UP_KEYWORDS	Lipid metabolism	54	3.988183161	5.28E-08
GOTERM_BP_DIRECT	GO:0006631~fatty acid metabolic process	30	2.215657312	1.19E-07
KEGG_PATHWAY	mmu01212:Fatty acid metabolism	16	1.1816839	3.02E-07
UP_KEYWORDS	Fatty acid metabolism	24	1.772525849	6.58E-07
GOTERM_BP_DIRECT	GO:0006629~lipid metabolic process	57	4.209748892	1.46E-06
GOTERM_BP_DIRECT	GO:0006633~fatty acid biosynthetic process	16	1.1816839	2.78E-05
KEGG_PATHWAY	mmu03320:PPAR signaling pathway	15	1.107828656	4.09E-04
UP_KEYWORDS	Fatty acid biosynthesis	11	0.812407681	0.001048132
KEGG_PATHWAY	mmu01040:Biosynthesis of unsaturated fatty acids	8	0.59084195	0.001103201
KEGG_PATHWAY	mmu00071:Fatty acid degradation	9	0.664697194	0.010678542
GOTERM_BP_DIRECT	GO:0006636~unsaturated fatty acid biosynthetic process	4	0.295420975	0.027838175
\				
**Annotation Cluster 7**	**GTPase activity**	**Enrichment Score: 4.181853442410703**		

Category	Term	Count	%	PValue
GOTERM_MF_DIRECT	GO:0003924~GTPase activity	40	2.954209749	1.00E-09
GOTERM_MF_DIRECT	GO:0005525~GTP binding	58	4.283604136	1.39E-09
UP_KEYWORDS	GTP-binding	50	3.692762186	1.75E-09
INTERPRO	IPR005225:Small GTP-binding protein domain	30	2.215657312	2.63E-07
UP_SEQ_FEATURE	nucleotide phosphate-binding region:GTP	44	3.249630724	4.52E-06
GOTERM_MF_DIRECT	GO:0019003~GDP binding	15	1.107828656	1.92E-05
INTERPRO	IPR027417:P-loop containing nucleoside triphosphate hydrolase	87	6.425406204	2.89E-05
GOTERM_BP_DIRECT	GO:0007264~small GTPase mediated signal transduction	30	2.215657312	5.82E-04
UP_KEYWORDS	Prenylation	18	1.329394387	0.010223033
UP_SEQ_FEATURE	short sequence motif:Effector region	14	1.033973412	0.01285581
INTERPRO	IPR001806:Small GTPase superfamily	16	1.1816839	0.017883478
INTERPRO	IPR020849:Small GTPase superfamily, Ras type	6	0.443131462	0.092287316
UP_SEQ_FEATURE	lipid moiety-binding region:S-geranylgeranyl cysteine	11	0.812407681	0.17917441
UP_SEQ_FEATURE	propeptide:Removed in mature form	14	1.033973412	0.782180297
\				
**Annotation Cluster 8**	**Oxidoreductase activity**	**Enrichment Score: 3.6368116796106165**		
Category	Term	Count	%	PValue
GOTERM_BP_DIRECT	GO:0055114~oxidation-reduction process	73	5.391432792	7.51E-06
GOTERM_MF_DIRECT	GO:0016491~oxidoreductase activity	60	4.431314623	5.29E-04
UP_KEYWORDS	Oxidoreductase	55	4.062038405	0.003094525
\				
**Annotation Cluster 9**	**Transcription regulation**	**Enrichment Score: 3.2380146999358397**		
Category	Term	Count	%	PValue
GOTERM_BP_DIRECT	GO:0006355~regulation of transcription, DNA-templated	198	14.62333826	1.16E-06
UP_KEYWORDS	Transcription	153	11.29985229	4.93E-06
UP_KEYWORDS	Transcription regulation	143	10.56129985	5.93E-05
GOTERM_BP_DIRECT	GO:0006351~transcription, DNA-templated	154	11.37370753	4.79E-04
UP_KEYWORDS	DNA-binding	95	7.016248154	0.452440958
GOTERM_MF_DIRECT	GO:0003677~DNA binding	118	8.714918759	0.507087523
\				
**Annotation Cluster 10**	**Respiratory chain**	**Enrichment Score: 3.203202459440069**		

Category	Term	Count	%	PValue
UP_KEYWORDS	Electron transport	23	1.698670606	2.16E-07
UP_KEYWORDS	Respiratory chain	13	0.960118168	2.54E-04
KEGG_PATHWAY	mmu05010:Alzheimer's disease	25	1.846381093	2.80E-04
KEGG_PATHWAY	mmu04932:Non-alcoholic fatty liver disease (NAFLD)	23	1.698670606	3.13E-04
KEGG_PATHWAY	mmu05012:Parkinson's disease	22	1.624815362	3.93E-04
KEGG_PATHWAY	mmu00190:Oxidative phosphorylation	20	1.477104874	0.001072875
KEGG_PATHWAY	mmu05016:Huntington's disease	25	1.846381093	0.001433721
GOTERM_CC_DIRECT	GO:0070469~respiratory chain	11	0.812407681	0.00288201
GOTERM_MF_DIRECT	GO:0008137~NADH dehydrogenase (ubiquinone) activity	8	0.59084195	0.006358906
GOTERM_CC_DIRECT	GO:0005747~mitochondrial respiratory chain complex I	9	0.664697194	0.010261158
UP_KEYWORDS	Ubiquinone	6	0.443131462	0.010631668
\				
**Annotation Cluster 11**	**Cell cycle**	**Enrichment Score: 3.0513120475475075**		
Category	Term	Count	%	PValue
UP_KEYWORDS	Cell cycle	69	5.096011817	3.75E-07
GOTERM_BP_DIRECT	GO:0007049~cell cycle	67	4.948301329	1.21E-05
UP_KEYWORDS	Cell division	36	2.658788774	0.002889163
GOTERM_BP_DIRECT	GO:0051301~cell division	37	2.732644018	0.007138631
GOTERM_BP_DIRECT	GO:0007067~mitotic nuclear division	25	1.846381093	0.072471944
UP_KEYWORDS	Mitosis	22	1.624815362	0.072686318
\				
**Annotation Cluster 12**	**protein kinase**	**Enrichment Score: 2.6892143859067703**		
Category	Term	Count	%	PValue
UP_KEYWORDS	Nucleotide-binding	175	12.92466765	5.29E-13
GOTERM_MF_DIRECT	GO:0000166~nucleotide binding	197	14.54948301	3.02E-12
UP_KEYWORDS	ATP-binding	121	8.93648449	1.95E-06
UP_KEYWORDS	Transferase	136	10.04431315	5.36E-06
GOTERM_MF_DIRECT	GO:0016740~transferase activity	124	9.158050222	2.92E-04
GOTERM_MF_DIRECT	GO:0005524~ATP binding	128	9.453471196	3.54E-04
UP_KEYWORDS	Kinase	58	4.283604136	0.006483469
GOTERM_MF_DIRECT	GO:0016301~kinase activity	58	4.283604136	0.013110458
GOTERM_BP_DIRECT	GO:0018105~peptidyl-serine phosphorylation	16	1.1816839	0.019760474

UP_KEYWORDS	Serine/threonine-protein kinase	34	2.511078287	0.029999489
UP_SEQ_FEATURE	nucleotide phosphate-binding region:ATP	78	5.76070901	0.035732122
GOTERM_MF_DIRECT	GO:0004674~protein serine/threonine kinase activity	37	2.732644018	0.049675546
GOTERM_BP_DIRECT	GO:0016310~phosphorylation	49	3.618906942	0.061652446
SMART	SM00220:S_TKc	31	2.289512555	0.076884921
INTERPRO	IPR011009:Protein kinase-like domain	43	3.17577548	0.084327303
GOTERM_MF_DIRECT	GO:0004672~protein kinase activity	42	3.101920236	0.098783044
UP_SEQ_FEATURE	active site:Proton acceptor	56	4.135893648	0.099649944
UP_SEQ_FEATURE	binding site:ATP	47	3.471196455	0.101355011
INTERPRO	IPR017441:Protein kinase, ATP binding site	31	2.289512555	0.118227306
GOTERM_BP_DIRECT	GO:0006468~protein phosphorylation	43	3.17577548	0.172264963
INTERPRO	IPR000719:Protein kinase, catalytic domain	37	2.732644018	0.210341314
INTERPRO	IPR008271:Serine/threonine-protein kinase, active site	25	1.846381093	0.222814306
UP_SEQ_FEATURE	domain:Protein kinase	38	2.806499261	0.239762492
\				
**Annotation Cluster 13**	**B and T cell homeostasis**	**Enrichment Score: 2.661611831517772**		
Category	Term	Count	%	PValue
GOTERM_BP_DIRECT	GO:0001782~B cell homeostasis	9	0.664697194	3.08E-04
GOTERM_BP_DIRECT	GO:0043029~T cell homeostasis	9	0.664697194	5.06E-04
GOTERM_BP_DIRECT	GO:0048536~spleen development	7	0.516986706	0.066372161
\				
**Annotation Cluster 14**	**DnaJ domain**	**Enrichment Score: 2.6303783295145013**		
Category	Term	Count	%	PValue
INTERPRO	IPR001623:DnaJ domain	11	0.812407681	9.86E-04
SMART	SM00271:DnaJ	10	0.738552437	0.002245917
UP_SEQ_FEATURE	domain:J	11	0.812407681	0.0034484
INTERPRO	IPR018253:DnaJ domain, conserved site	7	0.516986706	0.003942567
\				
**Annotation Cluster 15**	**circadian rhythm**	**Enrichment Score: 2.4301788330605674**		
Category	Term	Count	%	PValue
UP_KEYWORDS	Biological rhythms	19	1.403249631	3.20E-04
GOTERM_BP_DIRECT	GO:0048511~rhythmic	19	1.403249631	0.001063538

	process			
GOTERM_BP_DIRECT	GO:0032922~circadian regulation of gene expression	12	0.886262925	0.001500372
GOTERM_BP_DIRECT	GO:0042752~regulation of circadian rhythm	10	0.738552437	0.004676383
GOTERM_MF_DIRECT	GO:0001046~core promoter sequence-specific DNA binding	9	0.664697194	0.006385009
GOTERM_BP_DIRECT	GO:0007623~circadian rhythm	11	0.812407681	0.17194579
\				
**Annotation Cluster 16**	**Protein biosynthesis**	**Enrichment Score: 2.393365903873608**		
Category	Term	Count	%	PValue
GOTERM_BP_DIRECT	GO:0006413~translational initiation	13	0.960118168	1.04E-04
UP_KEYWORDS	Protein biosynthesis	22	1.624815362	1.29E-04
UP_KEYWORDS	Initiation factor	11	0.812407681	0.004271362
GOTERM_MF_DIRECT	GO:0003743~translation initiation factor activity	11	0.812407681	0.011748716
GOTERM_BP_DIRECT	GO:0001731~formation of translation preinitiation complex	6	0.443131462	0.017922032
BIOCARTA	m_eifPathway:Eukaryotic protein translation	4	0.295420975	0.038123658
KEGG_PATHWAY	mmu03013:RNA transport	18	1.329394387	0.038606828
\				
**Annotation Cluster 17**	**Ubiquitin conjugating enzyme activity**	**Enrichment Score: 2.2875065201924296**		
Category	Term	Count	%	PValue
KEGG_PATHWAY	mmu04120:Ubiquitin mediated proteolysis	23	1.698670606	7.01E-05
INTERPRO	IPR016135:Ubiquitin-conjugating enzyme/RWD-like	12	0.886262925	7.35E-04
GOTERM_MF_DIRECT	GO:0061631~ubiquitin conjugating enzyme activity	9	0.664697194	8.02E-04
INTERPRO	IPR000608:Ubiquitin-conjugating enzyme, E2	9	0.664697194	0.005148016
INTERPRO	IPR023313:Ubiquitin-conjugating enzyme, active site	7	0.516986706	0.008270018
UP_SEQ_FEATURE	active site:Glycyl thioester intermediate	12	0.886262925	0.017008786
GOTERM_BP_DIRECT	GO:0070936~protein K48-linked ubiquitination	7	0.516986706	0.078396043
GOTERM_BP_DIRECT	GO:0070534~protein K63-linked ubiquitination	5	0.369276219	0.21379371
\				

**Annotation Cluster 18**	**IFNy signaling**	**Enrichment Score: 2.245719658194264**		
Category	Term	Count	%	PValue
INTERPRO	IPR003191:Guanylate-binding protein, C-terminal	8	0.59084195	6.94E-06
INTERPRO	IPR015894:Guanylate-binding protein, N-terminal	8	0.59084195	1.23E-05
GOTERM_CC_DIRECT	GO:0020005~symbiont-containing vacuole membrane	4	0.295420975	0.015292408
GOTERM_BP_DIRECT	GO:0042832~defense response to protozoan	6	0.443131462	0.042111009
GOTERM_BP_DIRECT	GO:0071346~cellular response to interferon-gamma	9	0.664697194	0.062076509
GOTERM_BP_DIRECT	GO:0044406~adhesion of symbiont to host	3	0.221565731	0.126785567
GOTERM_BP_DIRECT	GO:0050830~defense response to Gram-positive bacterium	8	0.59084195	0.439693854
\\				
**Annotation Cluster 19**	**Cell red-ox homeostasis**	**Enrichment Score: 2.204614817771445**		
Category	Term	Count	%	PValue
GOTERM_BP_DIRECT	GO:0045454~cell redox homeostasis	13	0.960118168	5.55E-04
UP_KEYWORDS	Redox-active center	10	0.738552437	0.00147269
GOTERM_MF_DIRECT	GO:0003756~protein disulfide isomerase activity	6	0.443131462	0.006810197
UP_SEQ_FEATURE	domain:Thioredoxin	8	0.59084195	0.008971994
INTERPRO	IPR013766:Thioredoxin domain	8	0.59084195	0.010825789
INTERPRO	IPR017937:Thioredoxin, conserved site	5	0.369276219	0.016914131
INTERPRO	IPR012336:Thioredoxin-like fold	15	1.107828656	0.040382003
\\				
**Annotation Cluster 20**	**Protein deubiquitination**	**Enrichment Score: 2.179995156825928**		
Category	Term	Count	%	PValue
GOTERM_BP_DIRECT	GO:0071108~protein K48-linked deubiquitination	8	0.59084195	3.79E-04
GOTERM_MF_DIRECT	GO:0004843~thiol-dependent ubiquitin-specific protease activity	15	1.107828656	4.80E-04
GOTERM_MF_DIRECT	GO:0036459~thiol-dependent ubiquitinyl hydrolase activity	13	0.960118168	0.002101143
UP_KEYWORDS	Thiol protease	16	1.1816839	0.007319498
GOTERM_BP_DIRECT	GO:0016579~protein deubiquitination	11	0.812407681	0.02366881
GOTERM_MF_DIRECT	GO:0008234~cysteine-type peptidase activity	16	1.1816839	0.026801066

INTERPRO	IPR018200:Peptidase C19, ubiquitin carboxyl-terminal hydrolase 2, conserved site	8	0.59084195	0.039808582
INTERPRO	IPR001394:Peptidase C19, ubiquitin carboxyl-terminal hydrolase 2	8	0.59084195	0.051337672

Supplementary Table 6

List of primary antibodies used for protein expression analysis

Protein	Antibody cat. no	Dilution
HDAC3	sc-11417, Santa Cruz	1:500
ATGL	#21385, Cell Signaling	1:1000
ACADL	ab82853, Abcam	1:250
UCP1	ab10983, Abcam	1:1000
p293Ser-PDHE1α	ABS204, Merck Millipore	1:1000
p300Ser-PDHE1α	ABS194, Merck Millipore	1:1000
total PDHE1α	ab110330, Abcam	1:1500
ACLY	ab61762, Abcam	1:500
ACSS2	ab66038, Abcam	1:1000
HSP90α/β	sc-69703, Santa Cruz	1:500
α-TUBULIN	T9025, Sigma	1:5000
βACTIN	A5441, Sigma	1:5000
Histone H3	05-499, Upstate	1:500
H3K27ac	ab4729, Abcam	1:500
pan-acetyl H3	#43185, Cell Signaling	1:500
